# Surgical Antibiotic Prophylaxis Administration Improved after introducing Dedicated Guidelines: A Before-and-After Study from Dhulikhel Hospital in Nepal (2019–2023)

**DOI:** 10.3390/tropicalmed8080420

**Published:** 2023-08-18

**Authors:** Indira Shrestha, Sulekha Shrestha, Mathavaswami Vijayageetha, Pramesh Koju, Saugat Shrestha, Rony Zachariah, Mohammed Ahmed Khogali

**Affiliations:** 1 Dhulikhel Hospital, Kathmandu University Hospital, Dhulikhel 45200, Nepal; sulekhastha@gmail.com (S.S.); kojupramesh@gmail.com (P.K.); 2 Kathmandu University School of Medical Sciences, Dhulikhel 45200, Nepal; 3 PSG Institute of Medical Science and Research, Coimbatore 641004, India; drmvgeetha@gmail.com; 4 World Health Emergencies Programme, WHO Country Office, Kathmandu 44600, Nepal; shresthasau@who.int; 5 UNICEF, UNDP, World Bank, WHO Special Programme for Research and Training in Tropical Diseases (TDR), CH-1211 Geneva, Switzerland; zachariahr@who.int; 6 Institute of Public Health, College of Medicine and Health Sciences, United Arab Emirates University, Al Ain P.O. Box 17666, United Arab Emirates; ahmedm@uaeu.ac.ae

**Keywords:** health systems strengthening, operational research, SORT IT, surgical antibiotic prophylaxis, guidelines

## Abstract

(1) **Background**: Surgical antibiotic prophylaxis (SAP) is important for reducing surgical site infections. The development of a dedicated hospital SAP guideline in the Dhulikhel Hospital was a recommendation from a baseline study on SAP compliance. Compliance with this new guideline was enhanced through the establishment of a hospital committee, the establishment of an antibiotic stewardship program and the funding and training of healthcare professionals. Using the baseline and a follow-up study after introducing dedicated hospital SAP guidelines, we compared: (a) overall compliance with the SAP guidelines and (b) the proportion of eligible and non-eligible patients who received initial and redosing of SAP; (2) **Methods**: A before-and-after cohort study was conducted to compare SAP compliance between a baseline study (July 2019–December 2019) and a follow-up study (January 2023–April 2023); (3) **Results**: A total of 874 patients were in the baseline study and 751 in the follow-up study. Overall SAP compliance increased from 75% (baseline) to 85% in the follow-up study (*p* < 0.001). Over 90% of those eligible for the initial dose of SAP received it in both studies. Inappropriate use for those not eligible for an initial dose was reduced from 50% to 38% (*p* = 0.04). For those eligible for redosing, this increased from 14% to 22% but was not statistically significant (*p* = 0.272); (4) **Conclusions**: Although there is room for improvement, introduction of dedicated SAP guidelines was associated with improved overall SAP compliance. This study highlights the role of operational research in triggering favorable interventions in hospital clinical care.

## 1. Introduction

Antibiotic resistance, the ability of bacteria to resist the effect of antibiotics, is a public health problem worldwide [1,2,3]. On a global scale, resistant bacterial infections cause an estimated 700,000 deaths annually, and this number is expected to reach 10 million by 2050 [4]. Antibiotic resistance has considerable financial, economic and societal implications and risks the reversal of gains towards achieving the Sustainable Developmental Goals (SDGs) [5,6].

The main reason for the development and spread of antibiotic resistance is the inappropriate use of antibiotics. This includes irrational prescriptions; the incorrect choice, dose or duration of antibiotics; and the wrong route of administration [7].

In hospital settings, nearly 30–50% of antibiotics are used for surgical antibiotic prophylaxis (SAP) [8]. This prophylaxis is used to minimize the risk of surgical site infections (SSIs), which occur at incision sites of those undergoing surgery [9]. The appropriate use of SAP reduces the risk of SSIs and related morbidity and mortality [7]. Conversely, inappropriate SAP contributes to the development and spread of antibiotic resistance, and constitutes an economic burden on the healthcare system [10,11].

Guidelines to optimize the use of SAP [10] are essential to ensure appropriate prescription of antibiotics and are a fundamental component of good antimicrobial stewardship programs in hospitals [12,13]. The lack of guidelines possibly explained the low adherence to appropriate surgical antibiotic prophylaxis [14]. However, non-adherence to such guidelines remains a challenge in low- and middle-income countries (LMICs) [12,15], with 40% of SAP use deemed to be inappropriate [16].

In Nepal, the National Antibiotic Treatment Guidelines (NATGs) were developed in 2014, and these included guidance on SAP [17]. According to these guidelines, patients who undergo surgery, except those with clean wounds, must receive a dose of SAP intraoperatively (initial dose), followed by a repeat dose (redosing) when the surgery lasts for more than 2 h [18]. In 2020, a study conducted through the Structured Operational Research and Training Initiative (SORT IT) focusing on antimicrobial resistance (AMR) assessed compliance with the NATG for the administration of SAP at Dhulikhel Hospital [17]. The study revealed an overall compliance of 75%, in which 99% of those eligible for SAP received it, 50% of those not eligible for SAP received an unnecessary initial dose and 14% of those needing redosing did not receive it.

The attributed reason for these unfavorable findings was that the guidance on SAP administration was embedded as just one of many sections in the general guidelines for antibiotic treatment. Apathy among surgeons to read the full guidelines might have resulted in the SAP section being overlooked [17].

The findings of this first study [17] were effectively communicated to the management team at Dhulikhel Hospital. Consequently, a number of actions followed, which included: the establishment of a hospital committee for rational antibiotic use; the mobilization of funding for guidelines development and training; developing dedicated SAP guidelines for Dhulikhel Hospital; and training of all surgeons, anesthetists and nurses on the SAP guidelines. No prior studies were found in PubMed that had assessed the impact (before-and-after design) of introducing such measures on the appropriate use of SAP in Nepal. We decided to compare changes in the parameters of SAP compliance before and after the introduction of a dedicated SAP guideline and related interventions to improve compliance.

We aimed to describe the dissemination activities, recommendations and actions taken to enhance compliance (appropriate use) to SAP in patients who underwent surgery and the change in compliance. For assessing the impact on compliance, the study by Shrestha et al. [17] was considered the baseline study and the current study as the follow-up study. Henceforth, they are referred to as the baseline and follow-up study.

The specific objectives of this study were to compare compliance to SAP in the baseline study (July–December 2019) and the follow-up study (January–April 2023) after the introduction of dedicated SAP guidelines. The parameters that were compared included the: (a) overall compliance with the SAP guidelines and (b) the proportions of eligible and non-eligible patients who received initial dosing and redosing of SAP.

## 2. Materials and Methods

### 2.1. Study Design

A before-and-after cohort study was carried out to assess SAP compliance.

### 2.2. Study Setting

Nepal is a landlocked country located between India in the east, west and south and China in the north. It has a population size of 29.9 million people, of whom 21.5% reside in urban areas [19]. AMR is of major concern in Nepal, and the country has developed a national strategic plan for the use of antibiotics [20].

### 2.3. Study Site

Dhulikhel Hospital is a university referral hospital located 30 km east of Kathmandu. The hospital has 475 beds, and the Department of General Surgery has been offering high-quality medical care for pediatric, neurosurgery, cardiothoracic, vascular, gastrointestinal, and urology patients. All surgeons prescribe SAP. There was no protocol available for the administration of SAP at the hospital before conducting the baseline operational research study by Shrestha et al. [17] through the SORT IT program and before the findings were communicated to the hospital management team.

### 2.4. Dissemination Activities, Recommendations and Actions Taken

**Figure 1:**
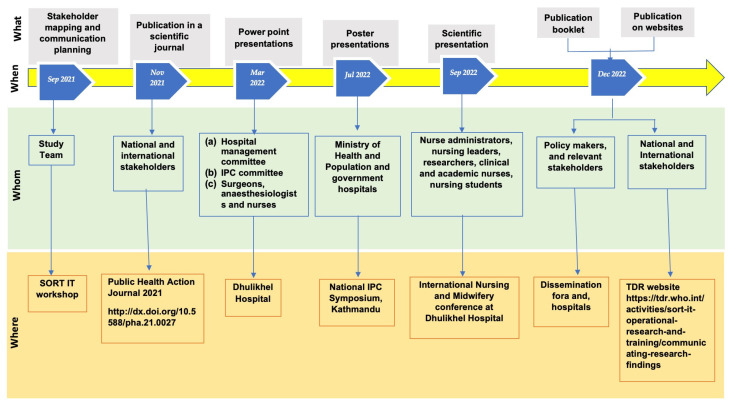
Dissemination activities of the baseline operational research study conducted on surgical antibiotic prophylaxis administration practices, Dhulikhel Hospital, Nepal, 2021. The Figure highlights: what was disseminated, when the dissemination happened, whom it addressed and where it was done [17]. Abbreviations: SORT IT—Structured Operational Research Training Initiative; TDR—the Special Programme for Research and Training in Tropical Diseases; WHO—World Health Organization; IPC—Infection Prevention and Control.

A specific SORT IT module was conducted in September 2021 to develop the practical skills and tools to effectively communicate the research findings [21]. These tools included: (1) a communication plan to target decision-makers and stakeholders; (2) a one-page plain language summary of the key messages (short and simple); (3) PowerPoint presentations to be used at national fora and conferences; and (4) an elevator pitch-a one-minute oral presentation for one-to-one conversations with decision makers. These tools were used to widen the opportunity for disseminating and communicating the research findings and make recommendations to the hospital management team in a non-technical language. To communicate the research findings of the baseline study and disseminate them effectively, we highlight what the communication activity was, when it was conducted, to whom it was targeted and where it was carried out (Figure 1).

Table 1 shows the recommendations, action status and details of actions for improving SAP use. This information was sourced from the published study, the plain language summary [22] and complemented by the study team of the baseline study who are also co-authors on the follow-up study.

### 2.5. Development of Dedicated Guidelines for SAP

The key recommendation was to develop dedicated guidelines for SAP in the hospital, and this was relayed to the hospital management team in March 2022. As a result, the hospital management team took a decision to establish a committee, consisting of members from the Pepartment of Surgery and Anesthesia and an infectious disease specialist in April 2022. By the end of 2022, through an iterative and consultative process, the committee was able to introduce dedicated guidelines for administering SAP in the hospital (Supplementary File S1).

The Special Programme for Research and Training in Tropical Diseases (TDR)’s SORT IT program provided the needed financial support for developing the guidelines and training the healthcare providers, all of which took place between July and December 2022. In contrast to the NATG, the dedicated SAP guidelines are user-friendly and provide clear guidance on the indications for the use of SAP; the choice and dosage of antibiotics, including alternative antibiotics for patients with a high risk of penicillin/cephalosporin allergy; and the timing of administering the initial dose and redosing of SAP.

### 2.6. Study Population and Periods

Both studies included all patients who underwent surgery from the Department of General Surgery during two study periods of July to December 2019 (baseline study period), and January to April, 2023 (follow-up study period).

### 2.7. Data Collection and Validation

Similar parameters were used to assess compliance during both the baseline and follow-up periods. Data on demographic characteristics, comorbidities, surgical site, wound classification and administration of SAP were gathered from patient medical records by data collectors using a paper-based data collection proforma. Two nurses were trained in data collection, especially for surgical wound classification and eligibility of SAP administration. The principal investigator supervised these nurses throughout the data collection period.

Data were double-entered into EpiData version 3.1 (EpiData Association, Odense, Denmark). The two files were then validated, and discordances were resolved by referring to the original sources of data.

### 2.8. Sample Size Calculation

The baseline study that assessed compliance to SAP in 2019 had shown an overall compliance of 75% using a sample of 874 consecutively enrolled patients who underwent surgery (baseline cohort). Following the implementation of interventions to improve SAP compliance, we estimated a 10% improvement in overall compliance (from 75% to 85%) by 2023. A minimal sample size of 375 patients was required each group (including the follow up cohort) to achieve a power of 90% and to detect a 10% absolute improvement in SAP compliance with a type I error rate of 5%. This calculation formed the basis of using a 3–4-month minimum recruitment period of consecutive patients for both study cohorts.

### 2.9. Data Analysis and Statistics

Data were analysed using EpiData Analysis (EpiData Association, Odense Denmark, version 2.2.2.183). Numbers and proportions of patients who received and did not receive SAP were calculated to assess the administration of antibiotics according to the type of surgical wound, eligibility for SAP, the correct timing of administration and correct redosing for patients in the baseline and follow-up periods (Table 2). The chi-squared test was used to compare differences in proportions, with *p* values < 0.05 considered statistically significant.

## 3. Results

### 3.1. Demographic and Clinical Characteristics of Surgical Patients

A total of 874 patients underwent surgery during the baseline study period and 751 patients underwent surgery during the follow-up study period. Table 3 shows the demographic and clinical characteristics of these patients. There were significant differences in both study populations, in terms of type of surgery, anatomical site of surgery, surgical wound class, comorbidity, and presence of prosthesis (*p* < 0.05).

### 3.2. Overall Proportion of Patients Who Received SAP in Compliance with the Guidelines

Overall SAP compliance increased from 75% in the baseline study to 85% in the follow-up study (*p* < 0.001, Table 4).

### 3.3. The Proportion of Eligible and Non-Eligible Patients Who Received Initial and Redosing of SAP

**Figure 2:**
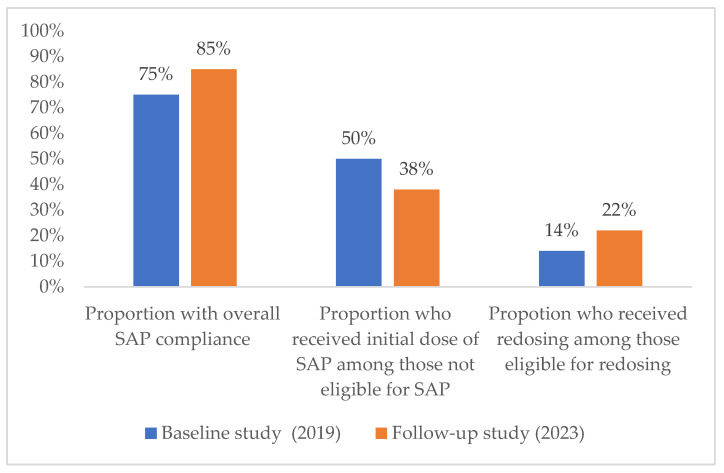
Changes in surgical antibiotic prophylaxis administration practices in the baseline (July–December 2019) and follow-up studies (January–April 2023) in the Department of General Surgery, Dhulikhel hospital, Nepal. Abbreviations: SAP—surgical antibiotic prophylaxis.

Table 4 shows the administration of the initial dose and redosing in patients who were eligible and not eligible for SAP. Ninety-nine percent (99%) and 95% of those eligible for the initial dose of SAP received it in the baseline and follow-up studies respectively and all (100%) received it at the correct time in both studies. For those not-eligible for an initial dose, 50% in the baseline study received it, and this was reduced to 38% in the follow-up study. For those eligible for redosing, this increased from 14% (baseline study) to 22% (follow-up study), although this change was not significantly different (*p* = 0.272). Two patients who were not eligible for redosing received it in the follow-up study compared to zero in the baseline study. Figure 2 illustrates the main findings graphically.

## 4. Discussion

This is the first study from Nepal that assessed changes in SAP administration practices after introducing dedicated hospital SAP guidelines and support activities to enhance their use. The study showed a significant increase (from 75% to 85%) in overall compliance with SAP guidelines, which is largely due to a reduction in the unnecessary administration of the initial SAP doses and an increase in the appropriate administration of SAP redosing.

This study shows the association between generating evidence through operational research and informing important decisions and actions for improving the rational use of antibiotics in patients undergoing surgery. While improving the quality of clinical care, this is also a step forward in enhancing the rational use of antibiotics, reducing unnecessary consumption and preventing the emergence of antibiotic resistance [23]. These preliminary findings are reassuring and laudable as they show that the hospital is on the right track towards galvanizing further efforts to increase SAP compliance. The experience from the Dhulikhel Hospital on “how to” enhance SAP compliance can be shared with other hospitals in the country.

The study strengths are that (a) we had large sample sizes and used the same parameters to assess compliance during both the baseline and follow-up periods, (b) the study theme is a national operational research priority and thus relevant to hospital practice and (c) the same trained team was involved with data collection and analysis, reducing the likelihood for bias. We also adhered to STROBE (Strengthening the Reporting of Observational Studies in Epidemiology) guidelines for the reporting of observational studies in epidemiology [24].

The main study limitation is that data collection for the follow-up study only started in January 2023 and the data were censored for analysis in April 2023. This was due to the tight timeline of funding for the follow-up study. Training of health workers on SAP was implemented in a phased manner, and there might also be a lag effect, which would tend to negate the overall impact seen in the follow-up period when compared to the baseline. As time goes on, this effect should dampen out, giving a more robust reflection of compliance. As there was no specific guidance regarding the choice and dose of drugs to be administered for SAP in the NATG, these two parameters were excluded from both studies. Although some patient characteristics differed between the baseline and follow-up studies, this should not have influenced the internal validity of the study as SAP eligibility assessments are independent of patient characteristics.

The study has a number of policy and practice implications. First, the overall level of compliance achieved in 2023 (85%) in the Dhulikhel Hospital is well above the 2% reported by Musmar et al. from Palestine [14], 21% by Shankar et al. from Nepal [25], 10.3% by Schmitt et al. from Brazil [26] and the 68% reported by Perulekar et al. from India [27]. In Dhulikhel, there was also a 10% increase (from 75% to 85%) between baseline and follow-up studies. The study finding is supported by So et al. from Canada who reported a significant improvement in compliance with the introduction of a guideline from 26.2% to 53.2% [28].

The inappropriate use of antibiotics for those not eligible for an initial dose was reduced from 50% to 38%. A reduction was also similarly reported by Segela et al. from 70.5% to 24.2% [29].

This begs the question “*Can the changes we observed in our current study be attributed to the introduction of dedicated SAP guidelines?*”. It is logical to think that when actions are specific and directed at improving a specific area of healthcare, there is likely to be positive change. This is an intuitive belief as a ‘favorable environment for change’ is induced when interventions such as the introduction of guidelines are followed up by training and backed up by a strong political commitment of decision makers. As there were no other parallel interventions that took place to possibly influence SAP compliance during the two assessments, we believe the observed improvements were associated with the package of interventions that followed the baseline study.

In our opinion, the enabling factors for the uptake of research findings included: research relevance, early involvement and buy-in of decision makers, which enhances co-ownership and responsibility; funding for the development of the guidelines and training of the healthcare providers; embedding research within the routine health system; and elaboration of dissemination activities.

Second, despite 85% SAP compliance being achieved in 2023, there is room for further improvement. There was a statistically significant decrease of 5% in the proportion of patients who were eligible for the initial dose and who received it. The reason for this is unknown and needs to be investigated. The specific focus should be on reducing the current proportion (37%) of those who receive the initial dose of SAP inappropriately and increasing the proportion of those eligible for redosing from 22%. The proportion eligible for redosing increased from 14% in the baseline study to 22%, but this favorable increase was not statistically significant. Further efforts to improve this parameter are needed and so too are further evaluations to monitor trends. Future research including qualitative evaluations may inform the way forwards. Bridging these gaps will have a direct effect on overall SAP compliance.

Third, a specific recommendation to get ‘a handle’ on trends in SAP compliance is to set up a quarterly routine monitoring system to assess SAP compliance for initial dosing and redosing. This will allow the monitoring and evaluation team to track SAP compliance and provide direct feedback to the hospital management and surgical teams. With a hospital committee now in place, there is a new window of opportunity for making further and sustained improvements in SAP compliance.

Finally, the experience from Dhulikhel Hospital provides the practical steps to assess and improve the practice of SAP administration in other hospitals across the country.

## 5. Conclusions

In conclusion, following the introduction of dedicated SAP guidelines and related support activities at the Dhulikhel Hospital, an overall improvement in SAP compliance was observed. While this improves the quality of clinical care, it also has a direct bearing on antibiotic stewardship and the emergence of antibiotic resistance [30]. This study also highlights the important role that operational research can play in informing decisions and triggering favorable interventions in hospital clinical care.

## Figures and Tables

**Table 1:** Recommendations, status and details of various actions stemming from the baseline operational research study for improving surgical antibiotic prophylaxis administration practices in Dhulikhel Hospital, Nepal, 2021 [17].

Recommendations	Action Status	Details of Action (When and What)
Establishment of a hospital committee for rational antibiotic use Establishment of an antibiotic stewardship program	Implemented Ongoing	*July 2022* Hospital committee established. Lead persons assigned from internal medicine and pharmacology. One doctor is being trained in infection, prevention and control
Develop a dedicated SAP guideline	Implemented	*May 2022* Seek funding for guidelines development and training. A proposal was accepted by the WHO country office in Nepal. 1500 US$ funding was provided by TDR.
		*December 2022* SAP guidelines developed and endorsed
Training of surgeons, anesthetists and nurses	Implemented	*December 2022* Training done in batches and continued.

Abbreviations: SAP—Surgical Antibiotic Prophylaxis; TDR—the Special Programme for Research and Training in Tropical Diseases; WHO—World Health Organization.

**Table 2:** Surgical wound class definition and indication (eligibility) for SAP, redosing and timing for initial dose of SAP according to dedicated SAP guidelines, Dhulikhel Hospital, Nepal, 2021.

Wound Class	Definition	Indication for SAP	Timing for Initial Dose of SAP	Indication for Redosing
Clean	Primarily closed, elective procedures involving no inflammation, no break in technique, and no entry into the gastrointestinal, oropharyngeal, biliary, genitourinary tracts or tracheobronchial tracts (e.g., herniorrhaphy)	Not recommended Recommended when: (1) risk factors are present, for example patients with immunosuppressive states, diabetes mellitus, malignancies or (2) patient has prosthesis in-situ	IV bolus: should be administered no more than 60 min prior to skin incision.	A single pre-operative dose is enough for most of the procedures, however, redosing is recommended when: (1) there is prolonged surgery, more than four hours from the time of the initial dose or (2) if major blood loss occurs (1500 mL)
Clean-contaminated	Surgery during which colonized viscus (e.g., gastrointestinal, tracheobronchial or genitourinary tract) is entered; minor breaches in technique; procedures following blunt trauma; cholecystectomy; prostate surgery; upper and/or lower urinary tract surgery; or uncomplicated appendectomy	Recommended		
Contaminated	Surgery in the presence of non-purulent inflammation or major spillage from a colonized viscus, major breach in aseptic technique, or traumatic wounds less than 4 h old	Recommended		
Dirty	Surgery in the presence of established infection (e.g., perforated viscous, devitalized tissue) and traumatic wounds more than 4 h old	NA *		

* Patients with dirty wounds are given therapeutic antibiotics before surgery and these patients do not qualify for SAP. Abbreviations: SAP—surgical antibiotic prophylaxis; NA—not applicable.

**Table 3:** Comparison of demographic and clinical characteristics of patients who underwent surgery in the baseline study (July–December 2019) and follow-up study (January–April 2023) in the Department of General Surgery, Dhulikhel Hospital, Nepal.

Characteristics	Baseline Study (July–December 2019)		Follow-Up Study (January–April 2023)		
	n	(%)	n	(%)	*p* Value
Total	874		751		
Sex					
Male	497	(57)	428	(57)	0.959
Female	377	(43)	323	(43)	
Age (years)					
Median [IQR]	40 (26–53)		43 (30–57)		0.432
Type of surgery					
Elective	661	(76)	638	(85)	<0.001
Emergency	213	(24)	113	(15)	
Anatomical site of surgery					
Gastrointestinal	476	(54)	339	(45)	<0.001
Inguinal Hernia	128	(15)	68	(9)	
Upper Urinary	101	(12)	100	(13)	
Lower Urinary	54	(6)	48	(7)	
Thoracic	8	(1)	8	(1)	
Vascular	42	(5)	88	(12)	
Others	65	(7)	100	(13)	
Surgical wound class					
Clean	202	(23)	216	(29)	0.001
Clean-contaminated	587	(67)	434	(58)	
Contaminated	57	(7)	70	(9)	
Dirty	28	(3)	31	(4)	
Comorbidity *					
None	817	(94)	692	(92)	0.002
Cancer	16	(2)	5	(0.7)	
HIV/AIDS	0	(0)	1	(0.1)	
TB	8	(1)	2	(0.3)	
Diabetes mellitus	31	(3)	51	(6.7)	
Presence/insertion of prosthesis					
No	758	(87)	694	(92)	<0.001
Yes	116	(13)	57	(8)	

* Multiple comorbidities are possible. Abbreviations: IQR—Interquartile range; HIV/AIDS—Human Immunodeficiency Virus/Acquired Immunodeficiency Syndrome; TB—Tuberculosis.

**Table 4:** Surgical antibiotic prophylaxis administration practices in the baseline study (July–December 2019) and follow-up study (January–April 2023) in the Department of General Surgery, Dhulikhel hospital, Nepal.

Characteristics	Baseline Study (July–December 2019)	Follow-Up Study (January–April 2023)	*p* Value *
	n (%)	n (%)	
Total patients	846	720	
Eligible for SAP	717	569	
Received initial dose	708 (99)	541 (95)	<0.001
Not eligible for SAP (a)	129	151	
Received initial dose (b)	65 (50)	57 (38)	0.045
Eligible for redosing (c)	164	27	
Received redosing (d)	23 (14)	6 (22)	0.272
Not eligible for redosing (e)	544	514	
Received redosing (f)	0	2 (0.4)	0.145
Overall SAP compliance *	632 (75)	612 (85)	<0.001

* Overall SAP compliance includes the total of: (i) those who were not eligible for SAP and did not receive SAP (a, b), (ii) those who were eligible for SAP redosing and received redosing (c, d), and (iii) those who were not eligible and did not receive redosing (e, f).

## Data Availability

Requests to access these data should be sent to the corresponding author.

## Supplementary Materials

The following supporting information can be downloaded at: https://www.mdpi.com/2414-6366/8/8/420/s1. S1. Surgical Antibiotics prophylaxis Guidelines.
