# Supplementary material for: Surgical Antibiotic Prophylaxis Administration Improved after introducing Dedicated Guidelines: A Before-and-After Study from Dhulikhel Hospital in Nepal (2019–2023)
Source: Trop Med Infect Dis. 2023 Aug 18;8(8):420. doi: 10.3390/tropicalmed8080420 (PMC10615196; doi:10.3390/tropicalmed8080420)
Supplement: Supplementary file 1 [file tropicalmed-08-00420-s001.zip › tropicalmed-2500668-supplementary.pdf]

SURGICAL ANTIBIOTIC PROPHYLAXIS GUIDELINE 2022

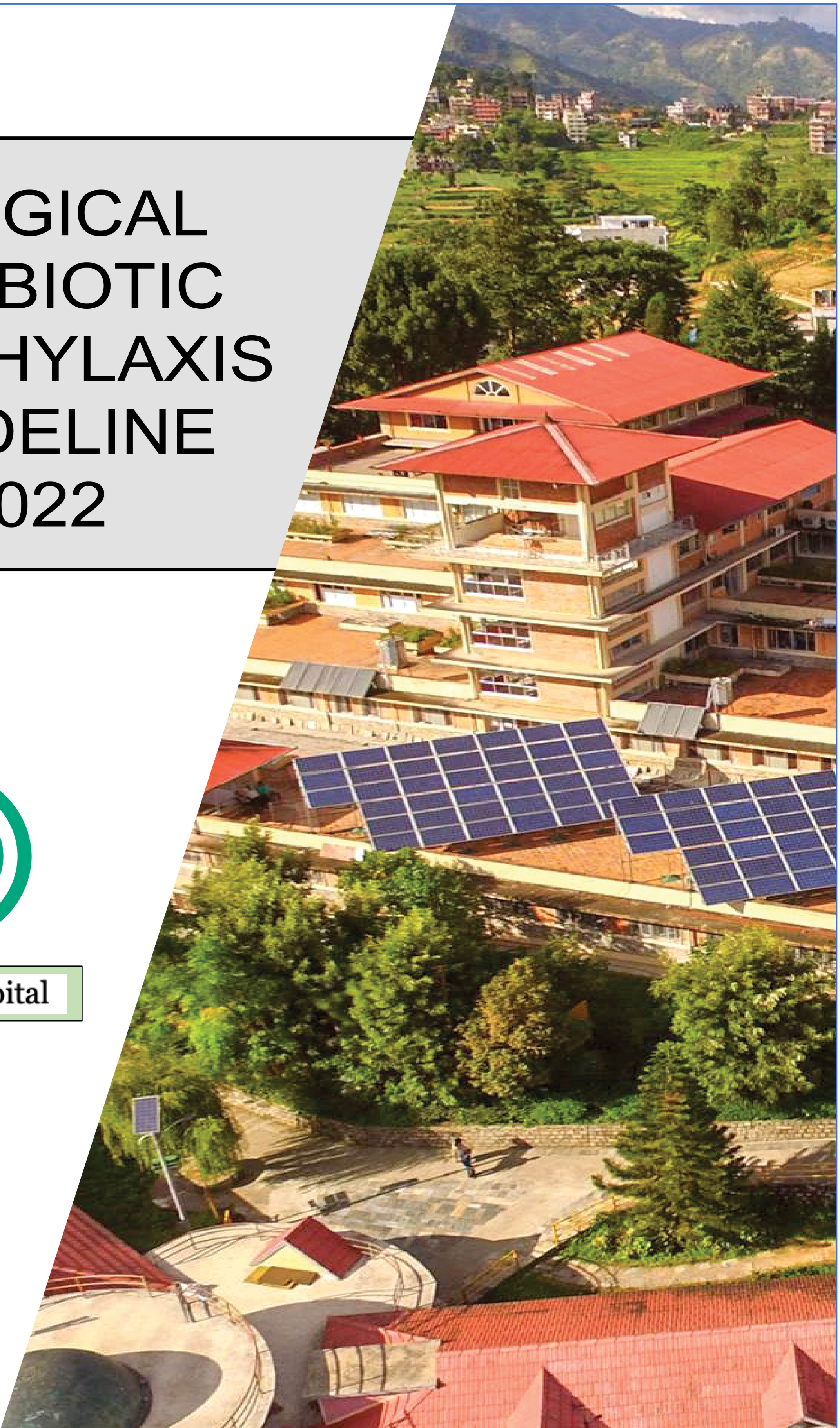

Dhulikhel Hospital

Surgical Antibiotic

P r o p h y l a x i s G u i d e l i n e

**Approved By**	**Administrative Director, Dhulikhel Hospital**
**Date Approved**	**25th December 2022**
**Author/Originator**	**Infection Prevention and Control Team**
**Name of Responsible Committee**	**Infection Prevention and Control Committee**
**Institution**	**Dhulikhel Hospital, Kathmandu University Hospital**

Forward

Surgical antibiotic prophylaxis consists of a brief course of antibiotics initiated preoperatively in order to decrease the risk of postoperative wound infections in the surgical patients.

The purpose of surgical antibiotic prophylaxis guideline is to help the health care providers to adhere to the best practice in using prophylactic antibiotics to surgical site infection in hospital.

The use of the guideline will also ensure the uniformity of practice in infection control in the hospital. I am confident that the judicial use of antibiotic as per the guideline will decrease the infection rate in the surgical patients and reduce the financial burden to the surgical patients and to the hospital.

Finally, I would like to convey my sincere thanks to the infection control team for their persistent effort and dedication in producing the guideline for prophylaxis use of antibiotics in controlling infection in hospital.

Thank you!

Prof. Dr. Bala Ram Malla

Medical Director, Dhulikhel Hospital

Advisors

Prof. Dr. Ram Kantha Makaju Shrestha
Executive Director, Dhulikhel Hospital

Prof. Dr. Rajendra Koju
Dean, Kathmandu University School of Medical Sciences (KUSMS)

Prof. Dr. Ramesh Makaju
Administrative Director, Dhulikhel Hospital

Prof. Dr. Dipak Shrestha
Associate Dean, Affiliated College, KUSMS

Prof. Dr. Bala Ram Malla
Medical Director, Dhulikhel Hospital
Associate Dean, Constitute Program, KUSMS

Associate Professor Dr. Ram Bahadur Gurung
Director, Hospital Services, Dhulikhel Hospital

Prof. Dr. Rajeev Shrestha
Chief, Research and Development
Head, Centre for Infectious Disease Research

Prof. Dr. Kunta Devi Pun
Nursing Director, Nursing and Midwifery Program, KUSMS

Associate Professor Ms. Subasna Shrestha
Director, Clinical Nursing, Dhulikhel Hospital

Guideline Committee Members List

Dr. Dipesh Tamrakar
Assistant Professor, Department of community medicine/infectious diseases

Dr. Pranodan Poudel
Medical officer, Research Assistant

Dr. Ashish Shrestha
Lecturer, Department of Anesthesiology and Critical care

Dr. Robin Man Karmacharya
Associate professor, Department of surgery

Dr. Sujan Makaju Shrestha
Lecturer, Department of surgery

Dr. Bibhusan Shrestha
Lecturer, Department of Surgery

Dr. Gonish Hada
Lecturer, Department of Surgery

Dr. Sabik Raj Kayastha
Lecturer, Department of Orthopedics and traumatology

Dr. Neeta Timilsina
Lecturer, Department of Obstetrics & Gynecology

Dr. Tina Shrestha
Lecturer, Department of Ophthalmology

Dr. Krishna Sundar Shrestha
Lecturer, Department of ENT & Head and Neck surgery

Dr. Pasanda Sharma
Lecturer, Department of Internal Medicine

Dr. Samjhana Basnet
Assistant Professor, Department of General Practice and Emergency Medicine

Mr. Surendra Kumar Madhup
Lecturer, Department of Clinical Microbiology

Mr. Hira Man Shankhadev
Senior Pharmacist
Head, Department of Pharmacy

Mr. Pramesh Koju
Public Health Officer, Department of Public Health and Community Programs

Ms. Sulekha Shrestha
Associate Professor, Infection control nurse

Ms. Jeny Shrestha
Nursing officer, Infection control nurse

Dhulikhel Hospital, Kathmandu University Hospital

Acknowledgements

Dhulikhel hospital, infection prevention and control (IPC) team would like to acknowledge the valuable contributions of all stakeholders who participated in the review and update of this surgical antibiotic treatment (SAP) guideline, for the health care professionals.

The Surgical Antibiotic Prophylaxis Guideline was made possible through the unreserved commitment of professionals in the meetings and workgroups during the review and update of the guideline. We wish to acknowledge the contributions of:

Structured Operational Research and Training Initiative (SORT IT) on tackling antimicrobial resistance (AMR, AMR-SORT IT)/World Health Organization, TDR.

The multidisciplinary team of health professionals for their valuable comments/suggestions e.g.

- Department of anesthesiology and critical care
- Department of gastrointestinal and general surgery, urosurgery, neurosurgery, cardiothoracic and vascular surgery
- Department of orthopedics and traumatology
- Department of obstetrics and gynecology
- Department of Ear, nose, throat (ENT), head and neck surgery
- Department of ophthalmology
- Department of oral and maxillofacial surgery
- Department of microbiology, pharmacology, internal medicine and community medicine

World Health Organization country office, Nepal for sponsoring the project of the guideline development/consultancy

Head of department of all surgical departments, anesthesiology and critical care for their continuous support and guidance for the development of the guideline

Dhulikhel hospital for the enormous support throughout the development of the guideline.

IPC Team, 2022

TABLE OF CONTENTS

Content **Page No.**

FORWARD

ACKNOWLEDGEMENTS

INTRODUCTION

GUIDELINE STATEMENT

ROLES AND RESPONSIBILITIES

POLICY REQUIREMENTS

IMPLEMENTATION AND MONITORING

ABBREBIATIONS

REFERENCES

APPENDICES

- **APPENDIX 1: BREAST PROCEDURES / ABDOMINAL PROCEDURES (INCLUDING SPLENECTOMY) / HERNIORRHAPY / INSERTION OF INFUSAPORT / CLEAN EXCISION PROCEDURE**
- **APPENDIX 2: CARDIOTHORACIC AND VASCULAR SURGERY (CTVS)**
- **APPENDIX 3: UROLOGICAL PROCEDURES**
- **APPENDIX 4: GASTROINTESTINAL SURGERY**
- **APPENDIX 5: NEUROSURGICAL PROCEDURES**
- **APPENDIX 6: EAR, NOSE, THROAT (ENT), HEAD AND NECK SURGERY**
- **APPENDIX 7: ORTHOPEDIC SURGERY (JOINT REPLACEMENT)**
- **APPENDIX 8: ORTHOPEDIC SURGERY (NON-JOINT REPLACEMENT)**
- **APPENDIX 9: OBSTETRICS AND GYNAECOLOGICAL SURGERY**
- **APPENDIX 10: OPHTHALMOLOGY SURGERY**
- **APPENDIX 11: ORAL AND MAXILLOFACIAL SURGERY**
- **APPENDIX 12: PREVENTION OF ENDOCARDITIS OR INFECTION OF PROSTHETIC IMPLANTS OR GRAFTS**

1. Introduction

Surgical antimicrobial prophylaxis refers to the use of antibiotics for the prevention of surgical site infections. Effective use of antibiotics to prevent infection is essential to reduce risks associated with surgical procedures. Efforts need to be made to maximize the quality of surgical antimicrobial prophylaxis prescribing.[1]

The key elements of appropriate surgical antibiotic prophylaxis prescribing include the correct indication, right antibiotic, drug dose, route, timing of administration and duration.[1]

2. Guideline Statement

Surgical antibiotic prophylaxis (SAP) is an integral part of surgical procedures to prevent the surgical site infections and improve the post-operative recovery process. This guideline has been formulated by the infection control committee at Dhulikhel hospital to aid the medical personnel with up-to-date information on proper antibiotic use, dosing, risks, contraindications, and post-operative care for the commonly performed surgical procedures at the institution mainly referring to the Australian guidelines.[2]

3. Role and Responsibilities

The SAP guideline applies to surgery performed in Dhulikhel hospital and all its outreach centers.

3.1 Infection control committee head is responsible for:

- ensuring clinicians have access to this guideline
- ensuring adequate resources are available to implement this guideline and the trainings are conducted as required
- maintaining effective mechanism for review of implementation of this guideline within the hospital

3.2 Infection control committee is responsible for:

- provision of governance over the use of prophylactic antibiotic agents in surgery
- collaborating with concerned departments for development, implementation, and update of surgical guidelines
- providing leadership for the training of clinical staff at Dhulikhel hospital on SAP administration

3.3 Prescribers are responsible for:

- safe and appropriate prescribing according to the general principles of SAP administration
- ensuring antibiotic administration within appropriate time frames as specified SAP guideline
- prescribing according to the appropriate surgical prophylaxis guideline
- where prescribing is not compliant with guidelines, properly documenting on the patient file
- ensuring patient and their caretakers get the proper information on their SAP prior to surgery

3.4 Pharmacists are responsible for:

- timely supply of antibiotic used in SAP guideline and their accountability

- safe, appropriate, and timely advice to prescribers and nurses with regards to the selection, dose, route, duration, and monitoring of antibiotic used in surgical prophylaxis

3.5 Nurses are responsible for:

- be up to date with the current SAP guideline and assist the prescribers to access the guideline
- ensuring safe and timely administration of prescribed antibiotic used in SAP guideline
- provision of department wise audit in relation to monitoring of antibiotic use as per the guideline
- assisting patients and caretakers to obtain information and understanding of their antibiotic therapy

4. Policy Requirements

4.1 Background

The surgical site infection (SSI) rate in Dhulikhel Hospital is 2.6% which is the lower than other centers in Nepal and places the hospital's infection control standards closer to the European Hospitals.[3] To maintain and further improve the SSI rates, a hospital-wide guideline on surgical antibiotic prophylaxis is necessary and this guideline on SAP thus aims to standardize the prescription of SAP across all the surgical departments.

4.2 Recommendations

Antibiotic prophylaxis should be considered where there is a clear indication, a risk of postoperative infection, or if postoperative infection will have serious consequences.[4]

In general, antibiotic prophylaxis is indicated in all procedures in the categories of “clean-contaminated”, “contaminated” or “dirty”. Prophylaxis for “clean” procedures is not generally indicated unless specific risk factors or circumstances that are associated with greater risk exist for example, patients of immunosuppressive states, diabetes mellitus, malignancies, prosthesis in-situ.

The recommended antibiotic prophylaxis regimens for specific surgical procedures, along with alternatives for patients with a high risk of penicillin/cephalosporin allergy, are available in appendices 1 to 12.

4.2.1 General Principles

Consider individual risk factors for every patient – In selected cases of immunosuppressive states, diabetics, allergic conditions, obese patients, prosthesis in-situ, remote infection, malignancies, or other pathologies the requirement of prophylaxis, choice of drug or dose may alter.

Pre-existing infections (known or suspected) – If such infections are present, appropriate treatment regime should be used instead of prophylactic medications for the procedure. Schedule the doses in a way that allows repeat dosing just prior to commencing skin incision.

4.2.2 Drug Administration

IV bolus: should be administered no more than 60 minutes prior to skin incision.[4] Administration outside of this time frame reduces effectiveness.[5]

IV infusion: start one hour before skin incision. This ensures adequate concentration at the time of incision and allows for any potential infusion-related toxicity to be recognized before induction.[2]

4.2.3 Methicillin-resistant *Staphylococcus aureus* (MRSA) Risk

When MRSA colonization is suspected (inpatient of unit with high risk of MRSA) for more than the last five days, OR MRSA infection is present, add Vancomycin.

4.2.4 Administration of vancomycin

Vancomycin 1g (1.5g for patients >80kg actual body weight) is administered by IV infusion started 30 minutes to 2 hours prior to skin incision. It should be given at a rate of 1g per hour (1.5g over 90 minutes).

Note: infusion can be completed after skin incision.

4.2.5 Administration of Clindamycin

Clindamycin 600mg (child: 15mg/kg up to 600mg) is administered by IV infusion over at least two hours just before the operation. For prolonged procedures, repeat four hourly intra-operatively.

4.2.6 Administration of Gentamicin

Dosing should be based on ideal body weight, provided that ideal body weight is less than the actual body weight.

4.2.7 Repeat Doses

A single pre-operative dose is enough for most of the procedures, however, for the following scenarios, repeat dosing is advisable:

- Prolonged surgery: More than four hours from the time of the first pre-operative dose when a short-acting agent is used (e.g., cefazolin).
- If major blood loss occurs, following fluid resuscitation. Major blood loss can be defined as follows:[6]
  - (i) blood loss exceeding circulating blood volume within a 24-hour period,
  - (ii) blood loss of 50% of circulating blood volume within a 3-hour period,
  - (iii) blood loss exceeding 150 ml/min, or
  - (iv) blood loss that necessitates plasma and platelet transfusion

When measuring the time to a second intraoperative dose, the interval from the time of the first preoperative dose rather than the surgical incision time should be used.

4.2.8 Obese patients

Consider increased dose of cefazolin (3g) if patient is obese (>120kg)

Consider increased dose of vancomycin (1.5g) for adult patients weighing more than 80kg

5. Classification of surgical wounds

Surgical procedures can be classified according to the level of microbial contamination routinely associated with that procedure and can be used to determine the need for, or choice of, antibiotic prophylaxis (see table 1 below). In general, antibiotic prophylaxis is indicated in all procedures in the categories of "clean-contaminated", "contaminated" or "dirty". Prophylaxis for "clean" procedures is not generally indicated unless specific risk factors or circumstances that are associated with greater risk or consequence of infectious complications exist.

Table 1: Classification of surgical wounds as defined by the CDC[7]

Classification of surgical wound	Criteria
Clean	Uninfected operative wounds in which no inflammation is encountered and the respiratory, gastrointestinal, genital or urinary tracts are not entered (and no break in aseptic technique). Clean wounds are primarily closed and, if necessary, drained with closed drainage. Operative incisional wounds that follow non-penetrating (blunt) trauma should be included in this category if they meet the criteria.
Clean-contaminated	Operative wounds in which the respiratory, gastrointestinal, genital (including female and male reproductive tracts), or urinary tracts are entered under controlled conditions and without unusual contamination. Specifically, operations involving the biliary tract, appendix, vagina, and oropharynx are included in this category, provided no evidence of infection or major break in technique is encountered.
Contaminated	Major breaks in sterile technique (e.g. open cardiac massage), gross spillage from the gastrointestinal tract, or the incision encounters acute, non-purulent inflammation (including necrotic tissue without evidence of purulent discharge). Open, fresh or accidental wounds are also included in this category.

Dirty (or infected)	Existing clinical infection or perforated viscera, as well as old traumatic wounds with retained devitalized tissue. This definition suggests that the organisms causing postoperative infection were present before the operation. In these cases, antibiotic treatment in addition to prophylaxis is required.

CDC = Centers for Disease Control and Prevention

6. **Implementation and Monitoring**

The infection control committee will coordinate the actions in response to the results of audits of antibiotic use in surgical prophylaxis. The results of annual audits should be reported to head of infection control committee along with a plan for continuous improvement. The guideline will be revised and updated annually by infection control team.

7. **Abbreviations**

CDC = Centers for Disease Control and Prevention
IV = Intravenous
MRSA = Methicillin-resistant *Staphylococcus aureus*
SAP = Surgical antibiotic prophylaxis
SSI = Surgical site infection

8. References

1. Ierano C, Nankervis JA, James R, Rajkhowa A, Peel T, Thursky K. Surgical antimicrobial prophylaxis. Australian prescriber. 2017 Dec;40(6):225.

2. Surgical Antimicrobial Prophylaxis Prescribing Guideline South Australian Health, v.3.0, 2021. Available from: https://www.sahealth.sa.gov.au/connect/policies (Last accessed on 25 Aug 2022).

3. Shrestha S, Wenju P, Shrestha R, et al. Incidence and Risk Factors of Surgical Site Infections in Kathmandu University Hospital, Kavre, Nepal. *Kathmandu Univ Med J (KUMJ)* 2016; 14: 107–111.

4. Bratzler DW, Dellinger EP, Olsen KM, et al. Clinical practice guidelines for antimicrobial prophylaxis in surgery. *Am J Health Syst Pharm* 2013; 70: 195–283.

5. Weber WP, Marti WR, Zwahlen M, et al. The timing of surgical antimicrobial prophylaxis. *Ann Surg* 2008; 247: 918–926.

6. Hellstern P, Haubelt H. Indications for plasma in massive transfusion. *Thrombosis Research* 2002; 107: S19–S22.

7. Onyekwelu I, Yakkanti R, Protzer L, Pinkston CM, Tucker C, Seligson D. Surgical wound classification and surgical site infections in the orthopaedic patient. Journal of the American Academy of Orthopaedic Surgeons. Global Research & Reviews. 2017 Jun;1(3).

9. Appendices

**Appendix 1**	Breast procedures / Abdominal procedures (including Splenectomy) / Herniorrhaphy / Insertion of infusaport / Clean excision procedures
**Appendix 2**	Cardiothoracic and Vascular Surgery (CTVS)
**Appendix 3**	Urological Procedures
**Appendix 4**	Gastrointestinal Surgery
**Appendix 5**	Neurosurgical Procedures
**Appendix 6**	Ear, Nose, Throat (ENT), Head and Neck Surgery
**Appendix 7**	Orthopedic Surgery (Joint Replacement)
**Appendix 8**	Orthopedic Surgery (Not-Joint Replacement)
**Appendix 9**	Obstetrics and Gynecology Surgery
**Appendix 10**	Ophthalmologic Surgery
**Appendix 11**	Oral and Maxillofacial Surgery
**Appendix 12**	Prevention of Endocarditis or Infection of Prosthetic Implants or Grafts

Surgical Antibiotic Prophylaxis Guideline

Appendix 1: Breast procedures / Abdominal procedures (including Splenectomy) / Herniorrhaphy / Insertion of infusaport / Clean excision procedure

PRE-OPERATIVE CONSIDERATIONS

Consider individual risk factors for every patient – In select cases of immunosuppressive states, diabetics, allergic conditions, obese patients, prosthesis in-situ, remote infection, malignancies, or other pathologies the requirement of prophylaxis, choice of drug or dose may alter.
Pre-existing infections (known or suspected) – If such infections are present, appropriate treatment regime should be used instead of prophylactic medications for the procedure. Schedule the doses in a way that allows repeat dosing just prior to commencing skin incision.

RECOMMENDED PRACTICE

Refer Section 3.2

RECOMMENDED PROPHYLAXIS[1,2]

Surgical Procedure	Recommended Prophylaxis	*High risk<br>Allergic to beta-lactams
**Uncomplicated clean procedures** (diagnostic excisional biopsy, stand-alone sentinel node biopsy, excision of scar tissue, lumpectomy (with or without needle or wire localization))<br><br>**Clean excision procedures**	Prophylaxis NOT recommended.	
**Breast**<br>Clean contaminated procedures (microdochectomy, mastectomy, reconstruction (incl. implants), reduction, sentinel node biopsy, re-operative surgery <6wks prior.	**Cefazolin 2g** IV (child: 30mg/kg up to 2g)<br>OR<br>**Cefuroxime 1.5g** IV<br><br>*High risk of MRSA infection:*<br>ADD Vancomycin 1g IV infusion (1.5g for patients with >80kg actual body weight)	Vancomycin 1g IV infusion (1.5g for patients with >80kg actual body weight)

Surgical Procedure	Recommended Prophylaxis	*High risk<br>Allergic to beta-lactams
Complicated clean-contaminated procedures<br><br>(prosthetic breast reconstruction surgery, prosthetic implant, autologous breast reconstruction surgery, breast augmentation surgery)	**Cefazolin 2g** IV<br>OR<br>**Cefuroxime 1.5g** IV<br><br>*High risk of MRSA infection:*<br>ADD **Vancomycin 1g** IV infusion (1.5g for patients with more than 80kg actual weight)<br><br>**POST-OPERATIVE:** For breast reconstruction or augmentation surgery, a further 2 doses of cefazolin (8 hours apart) can be considered	**vancomycin 1g** IV infusion (**1.5g** for patients more than 80kg **actual body weight**)<br><br>**POST-OPERATIVE:** For breast reconstruction or augmentation surgery, a single additional vancomycin dose 12 hour after the first dose can be considered
**Abdominal**<br>Procedures **involving viscera** (e.g., appendectomy, division of adhesions, resection)	**Metronidazole 500mg** IV infusion (child: 12.5 mg/kg),<br>PLUS, either,<br>**Cefazolin 2g** IV (child: 30mg/kg up to 2g)<br>OR<br>**Cefuroxime 1.5g** IV<br>OR<br>**Gentamicin 2mg/kg** IV<br><br>*High risk of MRSA infection:*<br>ADD **Vancomycin 1g** IV infusion (1.5g for patients with >80kg actual body weight)	**Metronidazole 500mg** IV infusion (child: 12.5 mg/kg),<br>PLUS,<br>**Gentamicin 2mg/kg** IV<br><br>*High risk of MRSA infection:*<br>ADD **Vancomycin 1g** IV infusion (1.5g for patients with >80kg actual body weight)
**Abdominal**<br>Procedures **not involving viscera** (e.g., abdominoplasty)	**Cefazolin 2g** IV (child: 30mg/kg up to 2g)<br>OR<br>**Cefuroxime 1.5gm** IV<br><br>*High risk of MRSA infection:*<br>ADD **Vancomycin 1g** IV infusion (1.5g for patients with >80kg actual	**Vancomycin 1g** IV infusion (1.5g for patients with >80kg actual body weight)

Surgical Procedure	Recommended Prophylaxis	*High risk<br>Allergic to beta-lactams
	body weight)	
**Splenectomy**<br>(Vaccination and post-splenectomy antibiotic prophylaxis is required in all the cases)	**Cefazolin 2g** IV (child: 30mg/kg up to 2g)<br>OR<br>**Cefuroxime 1.5g** IV<br><br>*High risk of MRSA infection:*<br>ADD **Vancomycin 1g** IV infusion (1.5g for patients with >80kg actual body weight)	**Vancomycin 1g** IV infusion (1.5g for patients with >80kg actual body weight)
**Other**<br>Insertion of infusaport/other devices	**Cefazolin 2g** IV (child: 30mg/kg up to 2g)<br>OR<br>**Cefuroxime 1.5g** IV<br><br>*High risk of MRSA infection:*<br>ADD **Vancomycin 1g** IV infusion (1.5g for patients with >80kg actual body weight)	**Vancomycin 1g** IV infusion (1.5g for patients with >80kg actual body weight)

POST-OPERATIVE CARE

Post-operative antibiotics are NOT advised unless an infection is confirmed or suspected apart from the conditions mentioned above.
If an infection is suspected, modify the antibiotic treatment based on clinical context and microbiological findings.

ABBREBIATIONS
IV = Intravenous
MRSA = Methicillin-resistant *Staphylococcus aureus*

REFERENCES

1. Surgical Antimicrobial Prophylaxis Prescribing Guideline South Australian Health, v.3.0, 2021. Available from: https://www.sahealth.sa.gov.au/connect/policies (Last accessed on 25 Aug 2022).

2. Berríos-Torres SI, Umscheid CA, Bratzler DW, et al. Centers for Disease Control and Prevention Guideline for the Prevention of Surgical Site Infection, 2017. *JAMA Surgery* 2017; 152: 784–791.

Surgical Antibiotic Prophylaxis Guideline

Appendix 2: Cardiothoracic and Vascular Surgery (CTVS)

PRE-OPERATIVE CONSIDERATIONS

Consider individual risk factors for every patient – In select cases of immunosuppressive states, diabetics, allergic conditions, obese patients, prosthesis in-situ, remote infection, malignancies, or other pathologies the requirement of prophylaxis, choice of drug or dose may alter.
Pre-existing infections (known or suspected) – If such infections are present, appropriate treatment regime should be used instead of prophylactic medications for the procedure. Schedule the doses in a way that allows repeat dosing just prior to commencing skin incision.
Local epidemiology - modify prophylaxis if there is a high local incidence of specific infections.

RECOMMENDED PRACTICE

Refer Section 3.2

RECOMMENDED PROPHYLAXIS[1]

Surgical Procedure	Recommended Prophylaxis	*High risk<br>Allergic to beta-lactams
**Coronary Artery Bypass Surgery (CABG)**<br><br>**Cardiac Valve Surgery**	**Cefazolin 2g** IV<br>OR<br>**Cefuroxime 1.5g** IV<br><br>Give **Cefazolin 2g** IV 8-hourly for another 2 doses commencing 8 hours after the first dose<br><br>*High risk of MRSA infection:*<br>**ADD vancomycin 1g** IV infusion (**1.5g** for patients more than 80kg **actual body weight)**	**Vancomycin 1g** IV infusion (**1.5g** for patients more than 80kg **actual body weight**)<br><br>PLUS<br><br>**gentamicin 5mg/kg** IV (single dose only – do not give postoperative dose)<br><br>Check kidney function first – if CrCl > 40 mL/min, give 1 additional dose of **vancomycin 1g** IV infusion (1.5g for patients more than 80kg actual body weight) 12 hours after the first dose
	Postoperative doses can be considered for all cardiac procedures for up to	

Surgical Procedure	Recommended Prophylaxis	*High risk<br>Allergic to beta-lactams
	24 hours	
**Atrial Septal Defect (ASD) closure, Patent Foramen Ovale (PFO) closure, Left Atrial Appendage Closure**<br><br>**Valvuloplasty, septal occlusion for high risk patients only** (e.g. femoral catheter > 6hrs, prosthetic valves, past history of endocarditis)	**Cefazolin 2g** IV<br>OR<br>**Cefuroxime 1.5g** IV<br>PLUS<br>**Vancomycin 1g** IV infusion (1.5g for patients more than 80kg actual body weight)	**Vancomycin 1g** IV infusion (1.5g for patients more than 80kg actual body weight)<br><br>PLUS<br><br>**Gentamicin 5mg/kg** IV
**Radio-frequency Ablation (RFA) for varicose veins**	Prophylaxis is NOT recommended.	
**RFA for varicose veins and venous ulcer**<br><br>**Peripheral bypass for peripheral arterial disease**<br><br>**Arterio-venous (AV) fistula creation**	**Cefazolin 2g** IV<br>OR<br>**Cefuroxime 1.5g** IV<br><br>*High risk of MRSA infection:*<br>ADD **Vancomycin 1g** IV infusion (1.5g for patients with more than 80kg actual weight)	**Vancomycin 1g** IV infusion (1.5g for patients with >80kg actual body weight), may repeat 12 hours after initial dose
**Vascular Reconstruction**	**Cefazolin 2g** IV (child: 30mg/kg up to 2g) repeated 8-hourly for two further doses post-operatively<br>OR<br>**Cefuroxime 1.5g** IV<br><br>*High risk of MRSA infection:*<br>ADD **Vancomycin 1g** IV infusion (1.5g for patients with more than 80kg actual weight)	**Vancomycin 1g** IV infusion (1.5g for patients with >80kg actual body weight), may repeat 12 hours after initial dose
**AV fistula revision or AV fistula with insertion of prosthetic material (e.g., Dacron graft)**	**Cefazolin 2g** IV<br>OR<br>**Cefuroxime 1.5gm** IV	**Vancomycin 1g** IV infusion (1.5g for patients with >80kg actual body weight)

Surgical Procedure	Recommended Prophylaxis	*High risk<br>Allergic to beta-lactams
	<u>*High risk of MRSA infection:*</u><br>ADD **Vancomycin 1g** IV infusion (1.5g for patients with more than 80kg actual weight)	
**Amputation of ischemic limb**	**Cefazolin 2g** IV (child: 30mg/kg up to 2g) repeated 8-hourly for two further doses post-operatively<br>OR<br>**Cefuroxime 1.5g** IV<br>PLUS,<br>**Metronidazole 500mg** IV infusion (child: 12.5 mg/kg up to 500mg) repeated 12 hours after initial dose<br><br><u>*High risk of MRSA infection:*</u><br>ADD **Vancomycin 1g** IV infusion (1.5g for patients with more than 80kg actual weight)	**Vancomycin 1g** IV infusion (1.5g for patients with >80kg actual body weight), repeated 12 hours after initial dose<br>PLUS,<br>**Metronidazole 500mg** IV infusion (child: 12.5 mg/kg up to 500mg) repeated 12 hours after initial dose
**Lobectomy / Pneumonectomy / Bullectomy / Peripheral bypass for peripheral arterial disease**	**Cefazolin 2g IV**<br>OR<br>**Cefuroxime 1.5g** IV<br>*If anaerobic cover required (empyema or abscess) then* ADD:<br><br>**Metronidazole 500mg** IV infusion<br>THEN<br><br>**Metronidazole 500mg** IV infusion for 1 more dose commencing 12 hours after the initial dose<br><br><u>*High risk of MRSA infection:*</u><br>ADD **Vancomycin 1g** IV infusion (1.5g for patients with more than 80kg actual weight)	**Vancomycin 1g** IV infusion (**1.5g** for patients more than 80kg **actual body weight**)<br><br>THEN<br><br>**Vancomycin 1g** IV infusion (**1.5g** for patients more than 80kg **actual body weight**) for 1 more dose commencing 12 hours after the initial dose<br><br>*If anaerobic cover required (empyema or abscess) then*<br><br>ADD **Metronidazole 500mg** IV infusion<br><br>THEN<br><br>**Metronidazole 500mg** IV

Surgical Procedure	Recommended Prophylaxis	*High risk<br>Allergic to beta-lactams
		infusion for 1 more dose commencing 12 hours after the initial dose
**Decortication/ Pleurectomy**	**Cefazolin 2g** IV<br>OR<br>**Cefuroxime 1.5g** IV<br><br>*If anaerobic coverage is needed ADD:*<br>**Metronidazole 500mg** IV infusion (child: 12.5 mg/kg)<br><br>*High risk of MRSA infection:*<br>ADD **Vancomycin 1g** IV infusion (1.5g for patients with >80kg actual body weight)	**Vancomycin 1g** IV infusion (1.5g for patients with >80kg actual body weight)<br><br>*If anaerobic coverage is needed ADD:*<br>**Metronidazole 500mg** IV infusion (child: 12.5 mg/kg)
Procedures involving insertion of prosthetic material<br><br>**Procedures associated with an increased risk of infection, including video-assisted thoracoscopic surgery (VATS), aneurysm repair, thromboendarterectomy, vein bypass, mediastinoscopy**	**Cefazolin 2g** IV<br>OR<br>**Cefuroxime 1.5g** IV<br><br>*High risk of MRSA infection:*<br>ADD **Vancomycin 1g** IV infusion (1.5g for patients with >80kg actual body weight)	**Vancomycin 1g** IV infusion (1.5g for patients with >80kg actual body weight)

POST-OPERATIVE CARE

Post-operative antibiotics are NOT advised unless an infection is confirmed or suspected apart from the conditions mentioned above.

If an infection is suspected, modify the antibiotic treatment based on clinical context and microbiological findings.

ABBREVIATIONS/DEFINITIONS

ASD = Atrial Septal Defect
AV = Arterio-venous
CABG = Coronary Artery Bypass Surgery
CTVS = Cardiothoracic and Vascular Surgery

IV = Intravenous
MRSA = Methicillin-resistant *Staphylococcus aureus*
PFO = Patent foramen ovale
RFA = Radio-frequency ablation
VATS = Video-assisted thoracoscopic surgery

REFERENCES

1. Surgical Antimicrobial Prophylaxis Prescribing Guideline South Australian Health, v.3.0, 2021. Available from: https://www.sahealth.sa.gov.au/connect/policies (Last accessed on 25 Aug 2022).

Surgical Antibiotic Prophylaxis Guideline

Appendix 3: Urological Procedures

PRE-OPERATIVE CONSIDERATIONS

Consider individual risk factors for every patient – In selected cases of immunosuppressive states, diabetics, allergic conditions, obese patients, prosthesis in-situ, remote infection, malignancies, or other pathologies the requirement of prophylaxis, choice of drug or dose may alter.
Pre-existing infections (known or suspected) – If such infections are present, appropriate treatment regime should be used instead of prophylactic medications for the procedure. Schedule the doses in a way that allows repeat dosing just prior to commencing skin incision.
Pre-operative urine screening – Urinary tract infections (UTIs) should be excluded or treated prior to surgery wherever possible. If surgery is urgent in the presence of confirmed infection or bacteriuria, use gentamicin 3mg/kg IV as a single preoperative dose. Higher doses may be required if systemic symptoms are present.

RECOMMENDED PRACTICE

The common organisms encountered are *Escherichia coli, Proteus mirabilis, Klebsiella spp., Enterococcus spp. Refer Section 3.2*

RECOMMENDED PROPHYLAXIS[1]

Surgical Procedure	Recommended Prophylaxis	*High risk<br>Allergic to beta-lactams
**Clean procedures**<br>**Open or laparoscopic urological procedure in when urinary tract not entered** (e.g., vasectomy, scrotal surgery, varicocele ligation) and prosthetic material is not implanted<br>**Extracorporeal shock-wave lithotripsy**<br><br>**Urodynamic studies**<br><br>**Diagnostic cystoscopy without manipulation of urinary tract**	Prophylaxis is NOT recommended.	

Surgical Procedure	Recommended Prophylaxis	*High risk<br>Allergic to beta-lactams
**Open/laparoscopic procedures when:**<br>> urinary tract entered<br>> urinary tract not entered but:<br>• patient is at risk of postoperative infection (e.g., urinary tract obstruction/ abnormalities)<br>• prosthetic material is inserted<br>OR,<br>• bacteriuria cannot be excluded<br>**Open Prostatectomy**	**Cefazolin 2g** IV<br>OR<br>**Cefuroxime 1.5g** IV<br><br>PLUS,<br>**Gentamicin 2mg/kg** IV (adults and children)<br>If risk of entry into bowel lumen, then ADD:<br>**Metronidazole 500mg** IV infusion (child: 12.5 mg/kg)<br><br>*High risk of MRSA infection:*<br>ADD **Vancomycin 1g** IV infusion (1.5g for patients with >80kg actual body weight)	**Vancomycin 1g** IV infusion (1.5g for patients with >80kg actual body weight)<br>PLUS,<br>**Gentamicin 2mg/kg** IV (adults and children)<br><br>If risk of entry into bowel lumen, then ADD:<br>**Metronidazole 500mg** IV infusion (child: 12.5 mg/kg),
**Endoscopic procedures**<br>➢ Calculi removal<br>➢ Extracorporeal Shock Wave Lithotripsy (ESWL) - only if high risk of infection<br>➢ Specific risk for postoperative infection	**Cefazolin 2g** IV<br>OR<br>**Cefuroxime 1.5g** IV<br><br>*Known urinary MRSA colonization:*<br>ADD **Vancomycin 1g** IV infusion (1.5g for patients with >80kg actual body weight)	**Gentamicin 2mg/kg** IV (adults and children)<br><br>*Known urinary MRSA colonization:*<br>ADD **Vancomycin 1g** IV infusion (1.5g for patients with >80kg actual body weight)
**Removal of calculi**<br>**Transurethral resection of prostate (TURP)**<br>**Stent Insertion**<br>**Ureteroscopy/instrumentation of upper tract** (including retrograde pyelogram)	**Gentamicin 2mg/kg** IV (adults and children)<br>*OR*<br>*(if gentamicin is contraindicated)*<br>**Cefazolin 2g** IV (child: 30mg/kg up to 2g)<br>OR<br>**Cefuroxime 1.5gm** IV<br><br>*Known urinary MRSA colonization:*	**Gentamicin 2mg/kg** IV (adults and children)<br><br>*OR (if gentamicin is contraindicated)*<br>**Trimethoprim 300mg** PO 1 hour prior to insertion

Surgical Procedure	Recommended Prophylaxis	*High risk<br>Allergic to beta-lactams
	ADD **Vancomycin 1g** IV infusion (1.5g for patients with >80kg actual body weight)	
**Transperineal prostatic biopsy**	**Cefazolin 2g** IV<br>OR<br>**Cefuroxime 1.5g** IV<br><br>*High risk of MRSA infection:*<br>ADD **Vancomycin 1g** IV infusion (1.5g for patients with >80kg actual body weight)	**Gentamycin 2mg/kg** IV<br>PLUS<br>**Vancomycin 1g** IV infusion (1.5g for patients with >80kg actual body weight)
**Transrectal prostatic biopsy**	**Ciprofloxacin 500mg** IV as a single dose	

POST-OPERATIVE CARE

Post-operative antibiotics are NOT advised unless an infection is confirmed or suspected apart from the conditions mentioned above.
If an infection is suspected, modify the antibiotic treatment based on clinical context and microbiological findings.

ABBREVIATIONS/DEFINITIONS

ESWL: = Extracorporeal Shock Wave Lithotripsy
IV = Intravenous
MRSA = Methicillin-resistant *Staphylococcus aureus*
PO = Per oral
TURP = Transurethral resection of prostate
UTI = Urinary Tract Infection

REFERENCES

1. Surgical Antimicrobial Prophylaxis Prescribing Guideline South Australian Health, v.3.0, 2021. Available from: https://www.sahealth.sa.gov.au/connect/policies (Last accessed on 25 Aug 2022).

Surgical Antibiotic Prophylaxis Guideline

Appendix 4: Gastrointestinal Surgery

PRE-OPERATIVE CONSIDERATIONS

Consider individual risk factors for every patient – In select cases of immunosuppressive states, diabetics, allergic conditions, obese patients, prosthesis in-situ, remote infection, malignancies, or other pathologies the requirement of prophylaxis, choice of drug or dose may alter.

Pre-existing infections (known or suspected) – If such infections are present, appropriate treatment regime should be used instead of prophylactic medications for the procedure. Schedule the doses in a way that allows repeat dosing just prior to commencing skin incision.

For patients with cardiac conditions refer to **Antibiotic Prophylaxis Guidelines for Prevention of Endocarditis** for further information.

RECOMMENDED PRACTICE

The common organism encountered are *Escherichia coli, Proteus mirabilis, Staphylococcus aureus, Pseudomonas aeruginosa, MRSA.*

Refer Section 3.2

RECOMMENDED PROPHYLAXIS[1]

Surgical Procedure	Recommended Prophylaxis	*High risk<br>Allergic to beta-lactams
**Hernia repair** with or without mesh insertion<br>**Transabdominal Preperitoneal (TAPP)** inguinal hernia repair	**Cefazolin 2g** IV<br>OR<br>**Cefuroxime 1.5g** IV<br>If entry into the bowel lumen is expected:<br><br>**ADD Metronidazole 500mg** IV infusion<br><br>*High risk of MRSA infection:*<br>ADD **Vancomycin 1g** IV infusion (1.5g for patients with more than 80kg actual weight)	**Vancomycin 1g** IV infusion (1.5g for patients more than 80kg actual body weight)<br><br>OR<br><br>If entry into the bowel lumen is expected give INSTEAD:<br><br>**Metronidazole 500mg** IV infusion PLUS **Gentamicin 2mg/kg** IV

Surgical Procedure	Recommended Prophylaxis	*High risk<br>Allergic to beta-lactams
**Gastrointestinal and esophageal**<br>Non-endoscopic procedures that enter the GI tract<br>OR<br>Non-endoscopic procedures that do not enter the GI lumen but patient has risk factors for postoperative infection	**Cefazolin 2g** IV infusion<br>OR<br>**Cefuroxime 1.5g** IV<br><br>*High risk of MRSA infection:*<br>ADD **Vancomycin 1g** IV infusion (1.5g for patients with more than 80kg actual weight)	**Gentamicin 2mg/kg** IV ^<br><br>PLUS<br><br>**Vancomycin 1g** IV infusion (1.5g for patients more than 80kg actual body weight)
**Small intestinal**<br>Non-endoscopic small intestinal procedures	**Cefazolin 2g** IV infusion<br>OR<br>**Cefuroxime 1.5g** IV<br><br>If the small intestine is obstructed:<br><br>**ADD metronidazole 500mg** IV infusion<br><br>*High risk of MRSA infection:*<br>ADD **Vancomycin 1g** IV infusion (1.5g for patients with more than 80kg actual weight)	**Gentamicin 2mg/kg** IV<br><br>PLUS<br><br>**Metronidazole 500mg** IV infusion<br><br>High risk of MRSA infection:<br><br>**Vancomycin 1g** IV infusion (1.5g for patients more than 80kg actual body weight)
**Colorectal**<br><br>Non-endoscopic colorectal procedures (e.g. colon resection, revision of anastomosis)<br><br>**Stoma**	**Cefazolin 2g** IV infusion<br>OR<br>**Cefuroxime 1.5g** IV<br>PLUS<br>**Metronidazole 500mg** IV infusion<br><br>*High risk of MRSA infection:*<br>ADD **Vancomycin 1g** IV infusion (1.5g for patients with more than 80kg actual weight)	**Gentamicin 2mg/kg** IV ^<br><br>PLUS<br><br>**Metronidazole 500mg** IV infusion<br><br>High risk of MRSA infection:<br><br>**Vancomycin 1g** IV infusion (1.5g for patients more than 80kg actual body weight
**Biliary Tract Surgery (including laparoscopic procedures)**<br><br>**Open cholecystectomy**	**Cefazolin 2g** IV infusion<br>OR<br>**Cefuroxime 1.5g** IV<br><br>*High risk of MRSA infection:*	**Gentamycin 2mg/kg** IV<br>PLUS<br>**Vancomycin 1g** IV infusion (1.5g for patients with >80kg actual body weight)

Surgical Procedure	Recommended Prophylaxis	*High risk<br>Allergic to beta-lactams
**Laparoscopic cholecystectomy** if patient has risk factors for postoperative infection (e.g., older than 70 years, diabetes, acute cholecystitis, non-functioning gallbladder)	ADD **Vancomycin 1g** IV infusion (1.5g for patients with more than 80kg actual weight)	
**Common bile duct (CBD) exploration**	**Cefotaxime 2g IV**<br>PLUS<br>**Metronidazole 500mg** IV infusion<br>PLUS<br>**Gentamycin 2mg/kg** IV	**Gentamycin 2mg/kg** IV<br>PLUS<br>**Vancomycin 1g** IV infusion (1.5g for patients with >80kg actual body weight)
**Pancreatic**<br>Whipple's procedure, pancreatic necrosectomy, pancreatectomy<br>**Liver resection**	**Cefazolin 2g** IV infusion<br>OR<br>**Cefuroxime 1.5g** IV<br>PLUS<br>**Metronidazole 500mg** IV infusion<br><br>*High risk of MRSA infection:*<br>ADD **Vancomycin 1g** IV infusion (1.5g for patients with more than 80kg actual weight)	**Gentamycin 2mg/kg** IV<br>PLUS<br>**Vancomycin 1g** IV infusion (1.5g for patients with >80kg actual body weight)

POST-OPERATIVE CARE

Post-operative antibiotics are NOT advised unless an infection is confirmed or suspected apart from the conditions mentioned above.

If an infection is suspected, modify the antibiotic treatment based on clinical context and microbiological findings.

ABBREVIATIONS/DEFINITIONS

CBD = Common Bile Duct

IV = Intravenous

MRSA = Methicillin Resistant *Staphylococcus aureus*

ERCP = Endoscopic Retrograde Cholangiopancreaticography

TAPP = Transabdominal Preperitoneal

REFERENCES

1. Surgical Antimicrobial Prophylaxis Prescribing Guideline South Australian Health, v.3.0, 2021. Available from: https://www.sahealth.sa.gov.au/connect/policies (Last accessed on 25 Aug 2022).

Surgical Antibiotic Prophylaxis Guideline

Appendix 5: Neurosurgical Procedures

PRE-OPERATIVE CONSIDERATIONS

Consider individual risk factors for every patient – In select cases of immunosuppressive states, diabetics, allergic conditions, obese patients, prosthesis in-situ, remote infection, malignancies, or other pathologies the requirement of prophylaxis, choice of drug or dose may alter.
Pre-existing infections (known or suspected) – If such infections are present, appropriate treatment regime should be used instead of prophylactic medications for the procedure. Schedule the doses in a way that allows repeat dosing just prior to commencing skin incision.
For patients with cardiac conditions refer to **Antibiotic Prophylaxis Guidelines for Prevention of Endocarditis** for further information.

RECOMMENDED PRACTICE

The common organism encountered are *Staphylococcus aureus, coagulase-negative staphylococci (CoNS), Staphylococcus epidermidis.*
Refer Section 3.2

RECOMMENDED PROPHYLAXIS

Surgical Procedure	Recommended Prophylaxis	*High risk<br>Allergic to beta-lactams
**Clean procedures**	Prophylaxis NOT recommended	
**Craniotomy procedures** for closed traumatic head injury<br>**Trans-sphenoidal procedures**<br>**Spinal procedures** (laminectomy, microscopic discectomy)<br>**External ventricular drain (EVD) placement**<br>**Microsurgery**<br>**Ventriculo-peritoneal/ventriculo-atrial shunt placement**<br>**Procedures involving insertion of prosthetic material**<br>**Decompressive hemicraniectomy for stroke cases or tumor cases**	**Flucloxacillin 1g** IV<br>OR<br>**Cefazolin 2g** IV<br>OR<br>**Cefuroxime 1.5g** IV<br><br>*High risk of MRSA infection:*<br>ADD **Vancomycin 1g** IV infusion (1.5g for patients with more than 80kg actual weight)	**Vancomycin 1g** IV infusion (1.5g for patients with more than 80kg actual weight)

Surgical Procedure	Recommended Prophylaxis	*High risk<br>Allergic to beta-lactams
**Re-exploration procedures**<br>**Burr hole drainage for chronic subdural hemorrhage**<br>**Peripheral nerve decompression**<br>**Neuronavigation guided brain biopsy**		
All clean contaminated procedures<br>**Sub labial resection of pituitary tumor**<br>**Endoscopic repair of CSF leak**<br>**Penetrating skull/ spine injuries**	**Cefuroxime 1.5g** IV then **Cefuroxime 750mg** every 8 hours<br>ADD<br>**Metronidazole 500mg** IV every 8 hours	**Vancomycin 1g** IV infusion (1.5g for patients with more than 80kg actual weight)

POST-OPERATIVE CARE

Post-operative antibiotics are NOT advised unless an infection is confirmed or suspected apart from the conditions mentioned above.
If an infection is suspected, modify the antibiotic treatment based on clinical context and microbiological findings.

ABBREVIATIONS/DEFINITIONS

CoNS = Coagulase Negative Staphylococci
CSF = Cerebrospinal Fluid
EVD = External Ventricular drain
IV = Intravenous
MRSA = Methicillin-resistant *Staphylococcus aureus*

Surgical Antibiotic Prophylaxis Guideline

Appendix 6: Ear, Nose, Throat (ENT), Head and Neck Surgery

PRE-OPERATIVE CONSIDERATIONS

Consider individual risk factors for every patient – In select cases of immunosuppressive states, diabetics, allergic conditions, obese patients, prosthesis in-situ, remote infection, malignancies, or other pathologies the requirement of prophylaxis, choice of drug or dose may alter.

Pre-existing infections (known or suspected) – If such infections are present, appropriate treatment regime should be used instead of prophylactic medications for the procedure. Schedule the doses in a way that allows repeat dosing just prior to commencing skin incision.

For patients with cardiac conditions refer to **Antibiotic Prophylaxis Guideline for Prevention of Endocarditis** for further information.

RECOMMENDED PRACTICE

The common organisms encountered are *Staphylococcus aureus and Klebseilla pneumoniae.*
Refer Section 3.2

RECOMMENDED PROPHYLAXIS

Surgical Procedure	Recommended Prophylaxis	*High risk<br>Allergic to beta-lactams
**ENT Surgeries**<br><br>**Uncomplicated or minor clean procedures**<br>• Uncomplicated ear surgery including tympanoplasty (not infected), otoplasty<br>• Uncomplicated nose or sinus surgery	Prophylaxis is NOT recommended<br><br>Patients with specific cardiac conditions (e.g. prosthetic heart valve) undergoing these procedures require antibiotic prophylaxis for endocarditis	
**Hearing implant procedures including cochlear implant**	**Cefazolin 2g** IV<br>OR<br>**Cefuroxime 1.5g** IV	**Vancomycin 1g** IV infusion (1.5g for patients with more than 80kg actual weight)

Surgical Procedure	Recommended Prophylaxis	*High risk<br>Allergic to beta-lactams
**ENT Procedures**<br><br>• Nose and sinus surgeries including septoplasty and turbinoplasty, endoscopic procedures (microlaryngoscopy, panendoscopy)<br>• Stapedectomy<br>• Tonsillectomy<br>• Adenoidectomy	**Ampicloxacillin 1g** IV<br>OR<br>**Cefazolin 2g** IV<br><br>*<u>High risk of MRSA infection:</u>*<br>ADD **Vancomycin 1g** IV infusion (1.5g for patients with more than 80kg actual weight)	**Clindamycin 600mg** IV
**ENT Procedures**<br><br>**Major ear surgery**<br>**Complex septorhinosplasty**<br>**Revision sinus surgery**	**Ampicloxacillin 1g** IV<br>OR<br>**Cefazolin 2g** IV<br>PLUS<br>**Metronidazole 500mg** IV<br><br>*<u>High risk of MRSA infection:</u>*<br>ADD **Vancomycin 1g** IV infusion (1.5g for patients with more than 80kg actual weight)	
**Tympanomastoid surgery**<br>**Laryngectomy (primary or salvage)**	**Ampicloxacillin 1g** IV<br>**OR**<br>**Cefazolin 2g** IV<br>PLUS<br>**Metronidazole 500mg** IV<br><br>*<u>High risk of MRSA infection:</u>*<br>ADD **Vancomycin 1g** IV infusion (1.5g for patients with more than 80kg actual weight)	**Clindamycin 600mg** IV<br>PLUS<br>**Gentamycin 2mg/kg** IV
	# Postoperative doses following laryngectomy can be considered but prophylaxis (intravenous or oral) should not continue beyond 24 hours.	
**Head and Neck Surgery**<br><br>**Thyroidectomy**	Prophylaxis is NOT recommended.	

Surgical Procedure	Recommended Prophylaxis	*High risk<br>Allergic to beta-lactams
**Simple lymph node excision (including submandibular lymph node excision) Parotidectomy**		
**Clean-contaminated procedures**<br><br>**Procedures involving insertion of prosthetic material**	**Cefazolin 2g** IV<br>OR<br>**Cefuroxime 1.5g** IV<br>If insertion through mucosal surfaces:<br>ADD **Vancomycin 1g** IV infusion (1.5g for patients with more than 80kg actual weight)	**Clindamycin 600mg** IV
**Extensive neck dissection for malignancy**<br><br>**Debulking or reconstructive surgery for malignancy**	**Cefazolin 2g** IV<br>OR<br>**Cefuroxime 1.5g** IV<br>**PLUS**<br>**Metronidazole 500mg** IV<br>*High risk of MRSA infection:*<br>ADD **Vancomycin 1g** IV infusion (1.5g for patients with more than 80kg actual weight)<br><br>**POST-OPERATIVE:** A further 2 doses of cefazolin (8 hours apart) and 1 dose of metronidazole (12 hours apart) may be considered. Prophylaxis should not extend beyond 24 hours.	**Clindamycin 600mg** IV<br>PLUS<br>**Gentamycin 2mg/kg** IV<br><br>**POST-OPERATIVE:** A further 2 doses of clindamycin (8 hours apart) may be considered. Prophylaxis should not extend beyond 24 hours.

POST-OPERATIVE CARE

Post-operative antibiotics are NOT advised unless an infection is confirmed or suspected apart from the conditions mentioned above.
If an infection is suspected, modify the antibiotic treatment based on clinical context and microbiological findings.

ABBREVIATIONS/DEFINITIONS

ENT = Ear, Nose, Throat
ESPAL = Endoscopic Sphenopalatine Artery Ligation
FESS = Functional endoscopic sinus surgery

I & D = Incision and Drainage
IV = Intravenous
MRM = Modified Radical Mastoidectomy
MRSA = Methicillin Resistant *Staphylococcus aureus*

Surgical Antibiotic Prophylaxis Guideline

Appendix 7: Orthopedic Surgery (Joint Replacement)

PRE-OPERATIVE CONSIDERATIONS

Consider individual risk factors for every patient – In select cases of immunosuppressive states, diabetics, allergic conditions, obese patients, prosthesis in-situ, remote infection, malignancies, or other pathologies the requirement of prophylaxis, choice of drug or dose may alter.
Pre-existing infections (known or suspected) – If such infections are present, appropriate treatment regime should be used instead of prophylactic medications for the procedure. Schedule the doses in a way that allows repeat dosing just prior to commencing skin incision.

RECOMMENDED PRACTICE

Refer Section 3.2

RECOMMENDED PROPHYLAXIS[1]

Surgical Procedure	Recommended Prophylaxis	*High risk<br>Allergic to beta-lactams
**Primary Total Hip Replacement (THR)**<br><br>**Total Knee Replacement (TKR)**	**Cefazolin 2g** IV<br>Then (postoperative):<br>Cefazolin 2g IV 8-hourly for up to two more doses<br>OR<br>**Cefuroxime 1.5g** IV<br><br>*High risk of MRSA infection:*<br>ADD **Vancomycin 1g** IV infusion (1.5g for patients with >80kg actual body weight)	**Vancomycin 1g** IV infusion (1.5g for patients with >80kg actual body weight)
**Patients requiring revision / re-operation (joint replacement)**	**Cefazolin 2g** IV<br>OR<br>**Cefuroxime 1.5g** IV<br>PLUS<br>**Vancomycin 1g** IV infusion (1.5g for patients more than 80kg actual body weight)	**Vancomycin 1g** IV infusion (1.5g for patients with >80kg actual body weight)<br><br>THEN (postoperative):<br>**Vancomycin 1g** IV infusion: single dose given 12 hours after initial dose

Surgical Procedure	Recommended Prophylaxis	*High risk<br>Allergic to beta-lactams
	THEN (postoperative):<br>**Cefazolin 2g** IV 8 hourly for a further 2 doses<br>PLUS<br>**Vancomycin 1g IV infusion:** single dose given 12 hours after initial dose	
	Note: **Pre-existing infections (known or suspected)** – if present, use appropriate treatment regimen instead of prophylactic regimen for procedure. Doses should be scheduled to allow for re-dosing just prior to skin incision.	
**Morcellised allografting at joint replacement**	Add approximately **250mg vancomycin** to bone cement for first femoral head equivalent, and 500mg for more than one head, and no more than 500mg to graft	
**Routine arthroscopic procedures**	Prophylaxis is NOT recommended<br>(Unless prosthesis is being inserted or patient is immunocompromised)	

POST-OPERATIVE CARE

Post-operative antibiotics are NOT advised unless an infection is confirmed or suspected apart from the conditions mentioned above.
If an infection is suspected, modify the antibiotic treatment based on clinical context and microbiological findings.

ABBREVIATIONS/DEFINITIONS

THR = Total Hip Replacement
TKR = Total Knee Replacement
IV = Intravenous
MRSA = Methicillin-resistant *Staphylococcus aureus*

REFERENCES

1. Surgical Antimicrobial Prophylaxis Prescribing Guideline South Australian Health, v.3.0, 2021. Available from: https://www.sahealth.sa.gov.au/connect/policies (Last accessed on 25 Aug 2022).

Surgical Antibiotic Prophylaxis Guideline

Appendix 8: Orthopedic Surgery (Non-joint Replacement)

PRE-OPERATIVE CONSIDERATIONS

Consider individual risk factors for every patient – In select cases of immunosuppressive states, diabetics, allergic conditions, obese patients, prosthesis in-situ, remote infection, malignancies, or other pathologies the requirement of prophylaxis, choice of drug or dose may alter.
Pre-existing infections (known or suspected) – If such infections are present, appropriate treatment regime should be used instead of prophylactic medications for the procedure. Schedule the doses in a way that allows repeat dosing just prior to commencing skin incision.

RECOMMENDED PRACTICE

The common organism encountered are *Staphylococcus aureus, Streptococcus, E. coli, Acinetobacter, Psuedomonas, MRSA*.
Refer Section 3.2

RECOMMENDED PROPHYLAXIS

Surgical Procedure	Recommended Prophylaxis	*High risk<br>Allergic to beta-lactams
**Spinal procedures**	**Cefazolin 2g** IV<br>OR<br>**Cefuroxime 1.5g IV**<br><br>*High risk of MRSA infection:*<br>ADD **Vancomycin 1g** IV infusion (1.5g for patients with >80kg actual body weight)	**Vancomycin 1g** IV infusion (1.5g for patients more than 80kg actual body weight)

**Internal fixation of fractures of large bones**<br>**Procedures involving insertion of prosthetic of allograft material**<br>**Other (closed) internal fixation**	**Cefazolin 2g** IV<br>OR<br>**Cefuroxime 1.5g** IV<br><br>*High risk of MRSA infection:*<br>ADD **Vancomycin 1g** IV infusion (1.5g for patients with >80kg actual body weight)	**Vancomycin 1g** IV infusion (1.5g for patients more than 80kg actual body weight)
**Lower limb amputation**	**Cefazolin 2g** IV If limb is ischemic:<br>OR<br>**Cefuroxime 1.5g** IV<br><br>ADD<br><br>**Metronidazole 500mg** IV infusion<br><br>*High risk of MRSA infection:*<br>ADD **Vancomycin 1g** IV infusion (1.5g for patients with >80kg actual body weight)	**Vancomycin 1g** IV infusion (1.5g for patients more than 80kg actual body weight)<br><br>PLUS<br><br>**Gentamicin 2mg/kg** IV (for procedures likely to continue for longer than 6 hours, consider using a 5mg/kg dose)<br><br>If limb is ischemic: ADD **Metronidazole 500mg** IV
**Open fractures - debridement + stabilization**	**Cefuroxime 1.5g** IV<br>ADD<br>**Gentamycin 160mg**<br>OR<br>**Crystalline penicillin 1 million unit IV**<br><br>*High risk of MRSA infection:*<br>ADD **Vancomycin 1g** IV infusion (1.5g for patients with >80kg actual body weight)	**Vancomycin 1g** IV infusion (1.5g for patients more than 80kg actual body weight)

POST-OPERATIVE CARE

Post-operative antibiotics are NOT advised unless an infection is confirmed or suspected apart from the conditions mentioned above.
If an infection is suspected, modify the antibiotic treatment based on clinical context and microbiological findings.

ABBREVIATIONS/DEFINITIONS

ORIF = Open Reduction Internal Fixation
CR = Closed Reduction
IV = Intravenous
MRSA = Methicillin-resistant *Staphylococcus aureus*

REFERENCES

1. Surgical Antimicrobial Prophylaxis Prescribing Guideline South Australian Health, v.3.0, 2021. Available from: https://www.sahealth.sa.gov.au/connect/policies (Last accessed on 25 Aug 2022).

Surgical Antibiotic Prophylaxis Guideline

Appendix 9: Obstetrics and gynecology Surgery

PRE-OPERATIVE CONSIDERATIONS

Consider individual risk factors for every patient – In select cases of immunosuppressive states, diabetics, allergic conditions, obese patients, prosthesis in-situ, remote infection, malignancies, or other pathologies the requirement of prophylaxis, choice of drug or dose may alter.
Pre-existing infections (known or suspected) – If such infections are present, appropriate treatment regime should be used instead of prophylactic medications for the procedure. Schedule the doses in a way that allows repeat dosing just prior to commencing skin incision.
Before hysterectomy – Screening for and treating bacterial vaginosis (BV) reduces BV-associated cuff infection.
Before surgical termination of pregnancy – Screening for and treating *Chlamydia, trachomatis* and BV reduces infectious complications.
Investigate patients for sexually transmitted infections (STIs) if they have symptoms of an STI or before insertion of an intrauterine device or before a transcervical procedure (including surgical termination of pregnancy and hysteroscopy). If the results of investigations are positive, provide appropriate treatment for the STI to reduce the risk of postprocedural infective complications. Ideally treatment should be completed before the procedure.

RECOMMENDED PRACTICE

The common organism encountered are *Escherichia coli* and *Klebsiella.*
Refer Section 3.2

RECOMMENDED PROPHYLAXIS[1]

Surgical Procedure	Recommended Prophylaxis	*High risk<br>Allergic to beta-lactams
**Gynaecological Procedures**<br>**Laparoscopic procedures that do not enter the bowel or vagina** (diagnostic, tubal sterilization, operative (except for hysterectomy))<br><br>**Other transcervical procedures** (cystoscopy, hysterectomy, IUCD	Prophylaxis NOT recommended.	

Surgical Procedure	Recommended Prophylaxis	*High risk<br>Allergic to beta-lactams
insertion, endometrial biopsy, D&C for non-pregnancy indication)		
**Hysterectomy**<br>**Gynaecological laparotomy procedures** (omentectomy, oophprectomy)<br>**Pelvic organ prolapse procedures**<br>**Mid-urethral sling procedures**<br>**Vaginal repair**	**Cefazolin 2g** IV<br>OR<br>**Cefuroxime 1.5g** IV<br><br>PLUS<br>**Metronidazole 500mg** IV infusion<br><br>*<u>High risk of MRSA:</u>*<br>Add **vancomycin 1g** IV infusion (1.5g for patients with more than 80kg actual body weight	**Clindamycin 600mg** IV infusion<br>PLUS,<br>**Gentamicin 2mg/kg** IV<br><br>*<u>High risk of MRSA:</u>*<br>Replace Clindamycin with **vancomycin 1g** IV infusion (1.5g for patients with more than 80kg actual body weight
**Obstetric Procedures**<br><br>**Lower Segment Cesarean Section (LSCS (elective or non-elective))**	**Cefazolin 2g** IV<br>OR<br>**Cefuroxime 1.5g** IV<br><br>*<u>High risk of MRSA:</u>*<br>**Add** cefazolin with **vancomycin 1g** IV infusion (1.5g for patients with more than 80kg actual body weight	**Clindamycin 600mg** IV infusion<br>PLUS,<br>**Gentamicin 2mg/kg** IV<br><br>*<u>High risk of MRSA:</u>*<br>**Replace** clindamycin with **vancomycin 1g** IV infusion (1.5g for patients with more than 80kg actual body weight)
**Assisted vaginal delivery**	**Amoxicillin+clavulanate (1+0.2g) IV (**as a single dose as soon as possible after assisted vaginal delivery, ideally within 6 hours)<br>**<u>Moderate risk penicillin allergy:</u>**<br>**Cefazolin 2g** IV **(**as a single dose as soon as possible after assisted vaginal delivery, ideally within 6 hours)<br>PLUS<br>**metronidazole 500mg** IV (as a single dose as soon as possible after assisted vaginal delivery, ideally within 6 hours)	**Clindamycin 600mg** IV infusion<br>PLUS<br>**Gentamicin 2 mg/kg** IV

Surgical Procedure	Recommended Prophylaxis	*High risk<br>Allergic to beta-lactams
**Surgical termination of pregnancy**	**Doxycycline 400mg** PO as a single dose (1 hour prior to the procedure)<br>OR<br>Azithromycin 1g PO (within 120 minutes before the procedure)	
**Prophylaxis for repair of obstetric anal sphincter injuries** (including third- or fourth-degree perineal tears)	**Cefazolin 2g** IV (as early as possible)<br>PLUS<br>**Metronidazole 500mg** IV infusion (as early as possible)<br><br>*<u>High risk of MRSA infection:</u>*<br>ADD **Vancomycin 1g** IV infusion (1.5g for patients with >80kg actual body weight)	**Clindamycin 600mg** IV infusion (as early as possible)
	Post-operative antibiotic therapy is recommended.	

POST-OPERATIVE CARE

Post-operative antibiotics are NOT advised unless an infection is confirmed or suspected apart from the conditions mentioned above.
If an infection is suspected, modify the antibiotic treatment based on clinical context and microbiological findings.

ABBREVIATIONS/DEFINITIONS

BV = Bacterial vaginosis
IUD = Intra-uterine device
IV = Intravenous
LSCS = Lower Segment Cesarean Section
MRSA = Methicillin resistant *Staphylococcus aureus*
PO = Per oral
STIs = Sexually transmitted infections

REFERENCES

1. Surgical Antimicrobial Prophylaxis Prescribing Guideline South Australian Health, v.3.0, 2021. Available from: https://www.sahealth.sa.gov.au/connect/policies (Last accessed on 25 Aug 2022).

Surgical Antibiotic Prophylaxis Guideline

Appendix 10: Ophthalmology Surgery

PRE-OPERATIVE CONSIDERATIONS

Consider individual risk factors for every patient – In select cases of immunosuppressive states, diabetics, allergic conditions, obese patients, prosthesis in-situ, remote infection, malignancies, or other pathologies the requirement of prophylaxis, choice of drug or dose may alter.
Pre-existing infections (known or suspected) – If such infections are present, appropriate treatment regime should be used instead of prophylactic medications for the procedure. Schedule the doses in a way that allows repeat dosing just prior to commencing skin incision.

RECOMMENDED PRACTICE

Refer Section 3.2

RECOMMENDED PROPHYLAXIS

Surgical Procedure	Recommended Prophylaxis	*High risk<br>Allergic to beta-lactams
All procedures	Pre-operatively: Immediately prior to surgical incision, apply sterile povidone-iodine 5% swab to conjunctival cul de sac, lid margins and periorbital skin and dry at 5 minutes. In patients with a povidone iodine (Betadine®) allergy, use a sterile product containing chlorhexidine acetate 0.05% for 5 minutes[1]	
Extra-ocular procedures	No strong evidence for IV prophylaxis.<br>For procedures where infection may be present, **Moxifloxacin 0.5%** eye drops four times a day post-operatively for 7 days. [2]	
Anterior intra-ocular procedures<br>• phacoemulsification / lens implant<br>• keratoplasty<br>• trabeculectomy / tube implant<br>• corneal graft	**Cefazolin 1mg/0.1ml** intracameral injection at the end of the procedure<br>PLUS,<br>**Ofloxacin 0.3%** eye drops two hourly post-operatively for one week	Intracameral moxifloxacin 0.5% can be a substitute of ceftazidime / cefazolin[3,4]<br>Intracameral vancomycin NOT recommended as it carries the risk of haemorrhagic occlusive retinal vasculitis[5]
Posterior intra-ocular procedures	**Ceftazidime 2.25 mg/0.1 mL** subconjunctival injection at the end of the procedure	

Surgical Procedure	Recommended Prophylaxis	*High risk<br>Allergic to beta-lactams
	PLUS,<br>**Ofloxacin 0.3%** eye drops two hourly post-operatively for one week	
**Skin graft**	**Ciprofloxacin 0.3%** Topical<br>OR<br>**Ofloxacin 0.3%** Topical<br>OR<br>**Levofloxacin 0.5%** Topical four doses every five minutes<br>PLUS,<br>Tab. Amoxicillin (500mg) + Clavulanate (125mg) PO	
**Orbital Procedures that communicate orbit and paranasal sinuses (orbital fracture or orbital decompression)**	If implant is used: Soak implant pre-operatively with antimicrobial solution<br>PLUS,<br>Tab. Amoxicillin (500mg) + Clavulanate (125mg) PO	
**Lacrimal sac + Nasolacrimal duct**	Intraoperative Tab. Amoxicillin (500mg) + Clavulanate (125mg) PO	

POST-OPERATIVE CARE

Post-operative use of topical antibiotics lack strong evidence.[2] A prolonged course of antibiotic drops or ointment is not advised unless an infection is known or suspected. Immunological defenses may be lowered in patients receiving prolonged topical steroid therapy or who received systemic steroid therapy prior to surgery, which may increase their risk of infection.[6] Chloramphenicol 0.5% eye drops can be taken four times daily for seven days if postoperative topical antibiotics are deemed required due to an increased risk of infection.[2] Due to a higher risk of resistance, tobramycin eye drops should only be used in individuals who have chloramphenicol hypersensitivity.[2] If an infection is suspected, modify the antibiotic treatment based on clinical context and microbiological findings.

ABBREVIATIONS/DEFINITIONS

IV = Intravenous
MRSA = Methicillin-resistant *Staphylococcus aureus*
PO = Per oral

REFERENCES

1. Merani R, McPherson ZE, Luckie AP, et al. Aqueous Chlorhexidine for Intravitreal Injection Antisepsis: A Case Series and Review of the Literature. *Ophthalmology* 2016; 123: 2588–2594.

2. Therapeutic Guidelines: Antibiotic, Surgical prophylaxis for ophthalmic surgery. 2014: Melbourne [Available at: www.tg.org.au].

3. Kessel L, Flesner P, Andresen J, et al. Antibiotic prevention of postcataract endophthalmitis: a systematic review and meta-analysis. *Acta Ophthalmol* 2015; 93: 303–317.

4. Zhou AX, Messenger WB, Sargent S, et al. Safety of undiluted intracameral moxifloxacin without postoperative topical antibiotics in cataract surgery. *Int Ophthalmol* 2016; 36: 493–498.

5. Miller MA, Lenci LT, Reddy CV, et al. Postoperative hemorrhagic occlusive retinal vasculitis associated with intracameral vancomycin prophylaxis during cataract surgery. *J Cataract Refract Surg* 2016; 42: 1676–1680.

6. Aronson, J., Meyler's Side Effects of Drugs (16th edition). 2016, Elsevier Science & Technology: Oxford, UK.

Surgical Antibiotic Prophylaxis Guideline

Appendix 11: Oral and Maxillofacial Surgery

PRE-OPERATIVE CONSIDERATIONS

Prophylaxis is not indicated for intra-oral procedures: dentoalveolar surgery (extractions, impactions, exposures); minor pathology (soft tissue, cysts).

Consider individual risk factors for every patient – In selected cases of immunosuppressive states, diabetics, allergic conditions, obese patients, prosthesis in-situ, remote infection, malignancies, or other pathologies the requirement of prophylaxis, choice of drug or dose may alter.

Pre-existing infections (known or suspected) – If such infections are present, appropriate treatment regime should be used instead of prophylactic medications for the procedure. Schedule the doses in a way that allows repeat dosing just prior to commencing skin incision.

For patients with cardiac conditions refer to **Antibiotic Prophylaxis Guidelines for Prevention of Endocarditis** for further information.

RECOMMENDED PRACTICE

The common organism encountered are *Staphylococcus, Streptococcus, Fusobacterium, Peptostreptococcus, Klebsiella, Pseudomonas, Porphyromonas.*

Refer Section 3.2

RECOMMENDED PROPHYLAXIS[1]

Surgical Procedure	Recommended Prophylaxis	*High risk<br>Allergic to beta-lactams
**Procedures involving insertion of dental implants**<br>**Clean procedures** (including dentoalveolar surgery (extractions, impactions, exposures, minor pathology soft tissue, cysts))	Prophylaxis not recommended	
**Procedures involving incision through the oral mucosa only**<br>**Orthognathic surgery**	**Benzyl penicillin 1.2g** IV (child < 12 years: 30mg/kg up to 1.2g)<br>Repeat dose 1-hourly intra-operatively	**Clindamycin 600mg** IV infusion (child: 15mg/kg up to 600mg)

Surgical Procedure	Recommended Prophylaxis	*High risk Allergic to beta-lactams
**Skin approach procedures (Oral cavity not involved)**<br>• Temporomandibular joint (arthrocentesis, reconstruction)<br>• Submandibular gland excision/removal<br>• Mandibular reconstruction (without bone graft*)*	**Cefazolin 2g** IV<br>OR<br>**Cefuroxime 1.5g** IV<br><br>*High risk of MRSA infection:*<br>ADD **Vancomycin 1g** IV infusion (1.5g for patients with >80kg actual body weight)	**Clindamycin 600mg** IV infusion
Skin and oral mucosa approach procedures (oral cavity involved)<br>• Orthognathic surgery (temporomandibular joint replacement)<br>• Sublingual gland excision and salivary gland procedures<br>• Intraoral bone grafting procedures<br>• Procedures involving insertion of prosthetic material	**Cefazolin 2g** IV<br>OR<br>**Cefuroxime 1.5g** IV<br>PLUS,<br>Metronidazole 500mg IV infusion<br><br>*High risk of MRSA infection:*<br>ADD **Vancomycin 1g** IV infusion (1.5g for patients with >80kg actual body weight)	**Clindamycin 600mg** IV infusion
	Postoperative doses can be considered following orthognathic surgery but prophylaxis (intravenous or oral) should not continue beyond 24 hours	
**Open reduction and internal fixation of mandibular fractures or midfacial fractures** (e.g. Le Fort or zygomatic*)*	**Cefazolin 2g** IV<br>OR<br>**Cefuroxime 1.5g** IV<br>PLUS,<br>Metronidazole 500mg IV infusion<br><br>*High risk of MRSA infection:*<br>ADD **Vancomycin 1g** IV infusion (1.5g for patients with >80kg actual body weight)	Clindamycin 600mg IV infusion
	Postoperative doses can be considered for high risk patients (e.g. fracture in tooth-bearing segment of the mandible, prolonged lag time between injury and surgery, a carious or unhealthy tooth left in	

Surgical Procedure	Recommended Prophylaxis	*High risk<br>Allergic to beta-lactams
	the fracture line, inability to surgically restore the mucosal barrier, extensive periodontal disease) but prophylaxis (intravenous or oral) should not continue beyond 24 hours	

POST-OPERATIVE CARE

Post-operative antibiotics are NOT advised unless an infection is confirmed or suspected apart from the conditions mentioned above.
If an infection is suspected, modify the antibiotic treatment based on clinical context and microbiological findings.

ABBREVIATIONS/DEFINITIONS

TMJ = Temporomandibular Joint
IV = Intravenous
ORIF = Open Reduction Internal Fixation
MRSA = Methicillin-resistant *Staphylococcus aureus*

REFERENCES

1. Surgical Antimicrobial Prophylaxis Prescribing Guideline South Australian Health, v.3.0, 2021. Available from: https://www.sahealth.sa.gov.au/connect/policies (Last accessed on 25 Aug 2022).

Surgical Antibiotic Prophylaxis Guideline

Appendix 12: Prevention of Endocarditis or Infection of Prosthetic Implants or Grafts

PRE-OPERATIVE CONSIDERATIONS

Antibiotic prophylaxis to prevent endocarditis is ONLY recommended for patients with cardiac conditions associated with the HIGHEST RISK of adverse outcomes from endocarditis (See Box 1) and only for certain conditions (See Box 2).

**Box 1: Cardiac conditions for which antibiotic prophylaxis to prevent endocarditis is recommended**
• Prosthetic cardiac valve or prosthetic material used for cardiac valve repair<br>• Previous infective endocarditis<br>• Cardiac transplantation with the subsequent development of cardiac valvulopathy<br>• Rheumatic heart disease in all procedures<br>• Congenital heart disease, only if it involves:<br>a) unrepaired cyanotic defects, including palliative shunts and conduits<br>b) completely repaired defects with prosthetic material or devices, whether placed by surgery or catheter intervention, during the first six months after the procedure (after which the prosthetic material is likely to have endothelialized)<br>OR<br>c) repaired defects with residual defects at, or adjacent to the site of a prosthetic patch or device (which inhibit endothelialisation).

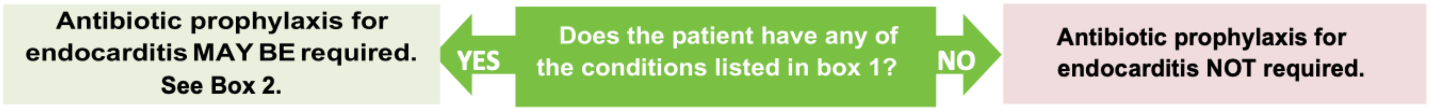

**Box 2: Procedures where antibiotic prophylaxis for endocarditis may or may not be required**	
**Prophylaxis ALWAYS REQUIRED**	**Prophylaxis IS NOT REQUIRED**
**DENTAL PROCEDURES:**<br>• tooth extractions<br>• periodontal procedures including surgery, subgingival scaling and root planning<br>• replanting avulsed teeth<br>• other surgical procedures (e.g., implant placement, apicoectomy)<br>**RESPIRATORY PROCEDURES:**<br>Any invasive procedure involving incision or biopsy of respiratory mucosa, for example:<br>• tonsillectomy/ adenoidectomy<br>• surgery involving bronchial, sinus, nasal or middle ear mucosa, including tympanostomy tube insertion.<br>**GENITOURINARY AND GASTROINTESTINAL PROCEDURES:** Any procedure where antibiotic prophylaxis is indicated for surgical reasons<br>• lithotripsy<br>• any genitourinary procedure in the presence of a genitourinary infection unless already treating enterococci (for elective cystoscopy or urinary tract manipulations, obtain a urine culture and treat any bacteruria beforehand)<br>• any gastrointestinal procedure in the presence of an intra- abdominal infection unless already treating enterococci<br>• sclerotherapy for oesophageal varices.<br>**OTHER PROCEDURES:**<br>• Incision and drainage of local abscess: brain, boils and carbuncles, dacryocystitis, epidural, lung, orbital, perirectal, pyogenic liver, tooth, surgical procedures through infected skin.	**DENTAL PROCEDURES:**<br>• oral examination<br>• infiltration and block local anaesthetic injection<br>• restorative dentistry<br>• supragingival rubber dam clamping and placement of rubber dam<br>• intracanal endodontic procedures<br>• removal of sutures<br>• impressions and construction of dentures<br>• orthodontic bracket placement and adjustment of fixed appliances<br>• application of gels<br>• intraoral radiographs<br>• supragingival plaque removal<br>**RESPIRATORY PROCEDURES:**<br>• endotracheal intubation<br>• rigid or flexible bronchoscopy with or without incision or biopsy<br>**GENITOURINARY AND GASTROINTESTINAL PROCEDURES:**<br>• urethral catheterisation, uterine dilatation and curettage, sterilization procedures, insertion or removal of intrauterine contraceptive device<br>• obstetric procedures<br>• transoesophageal echocardiography<br>• endoscopy (with or without gastrointestinal biopsy including colonoscopy)

• Percutaneous endoscopic gastrostomy	

RECOMMENDED PRACTICE

Refer Section 3.2

RECOMMENDED PROPHYLAXIS[1]

Surgical Procedure	Recommended Prophylaxis	*High risk<br>Allergic to beta-lactams
**Dental procedures**<br>**Tonsillectomy**<br>**Adenoidectomy**	**Amoxicillin 2g** PO (child: 50mg/kg up to 2g) 1 hour prior to the procedure<br><br>OR if oral administration not possible<br>**Amoxicillin 2g** IV (child: 50mg/kg up to 2g)	**Clindamycin 600mg** PO (child: 20mg/kg up to 600mg) 60 to 120 minutes prior to procedure<br>OR if oral administration not possible<br>**Clindamycin 600mg** IV (child: 20mg/kg up to 600mg)
**All other procedures**	**Amoxicillin 2g** IV (child: 50mg/kg up to 2g)	**Vancomycin 1g** IV infusion (1.5g for patients with more than 80kg actual body weight) (child: 30mg/kg up to 1.5g)

ABBREVIATIONS/DEFINITIONS

IV = Intravenous
MRSA = Methicillin-resistant *Staphylococcus aureus*
PO = Per oral

REFERENCES

1. Surgical Antimicrobial Prophylaxis Prescribing Guideline South Australian Health, v.3.0, 2021. Available from: https://www.sahealth.sa.gov.au/connect/policies (Last accessed on 25 Aug 2022).

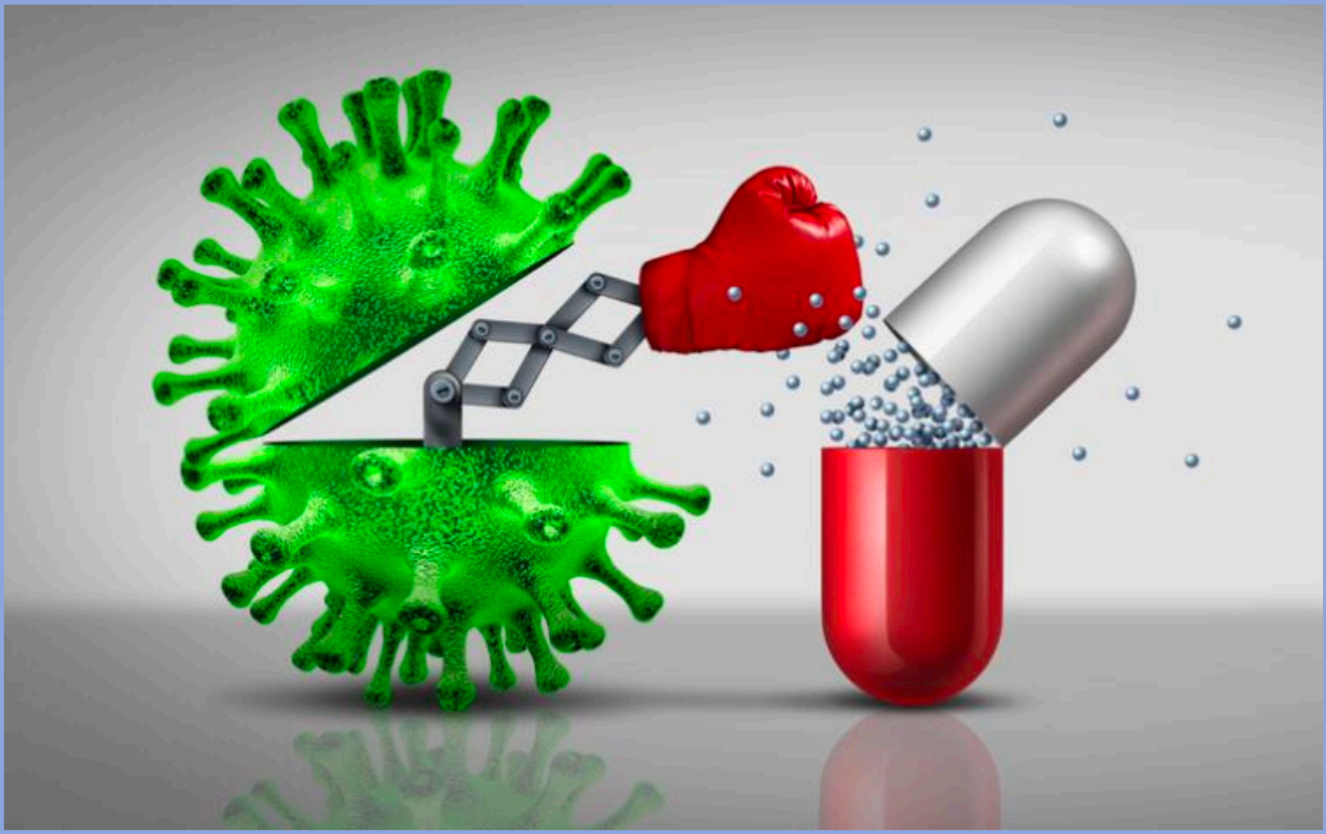

Antimicrobial Resistance; major threat to health and human development

Dhulikhel Hospital
Kathmandu University Hospital
Dhulikhel, Kavrepalanchowk, Nepal
P.O. Box: 11008
Phone No. +977-011-490497
Email: direct@dhulikhelhospital.org
Website: www.dhulikhelhospital.org
